# DNA Methylation Episignature as a Novel Diagnostic Tool for Diamond‐Blackfan Anemia Syndrome

**DOI:** 10.1002/ajh.70141

**Published:** 2025-11-17

**Authors:** Paola Quarello, Karim Karimi, Slavica Trajkova, Emanuela Garelli, Mehdi Samadieh, Emanuela Iovino, Tommaso Pippucci, Giovanni Papagni, Sandra Dalfonso, Lucia Corrado, Serena Rizzo, Adriana Carando, Jennifer Kerkhof, Jessica Rzasa, Haley McConkey, Michael Levy, Marco Zecca, Francesca Fioredda, Angelica Barone, Simone Cesaro, Maria Gabelli, Francesca Torchio, Giulia Zucchetti, Maria Elena Cantarini, Paola Corti, Ugo Ramenghi, Franco Locatelli, Franca Fagioli, Bekim Sadikovic, Alfredo Brusco

**Affiliations:** ^1^ Pediatric Onco‐Hematology, Stem Cell Transplantation and Cellular Therapy Division Regina Margherita Children's Hospital Turin Italy; ^2^ Department of Public Health and Pediatrics Sciences University of Torino Turin Italy; ^3^ Molecular Diagnostics Program, and Verspeeten Clinical Genome Centre London Health Sciences Centre London Ontario Canada; ^4^ Department of Neurosciences Rita Levi‐Montalcini University of Torino Turin Italy; ^5^ IRCCS Azienda Ospedaliero‐Universitaria of Bologna Bologna Italy; ^6^ Department of Health Sciences University of Piemonte Orientale UPO Novara Italy; ^7^ Medical Genetics Unit Città della Salute e della Scienza University Hospital Torino Italy; ^8^ Pediatric Hematology/Oncology, Fondazione IRCCS Policlinico “San Matteo” Pavia Italy; ^9^ Unit of Hematology‐IRCCS Istituto Giannina Gaslini Genoa Italy; ^10^ Pediatric Hematology Oncology Azienda Ospedaliero‐Universitaria Parma Italy; ^11^ Pediatric Hematology Oncology, Ospedale Donna Bambino Azienda Ospedaliera Universitaria Integrata Verona Italy; ^12^ Pediatric Hematology, Oncology and Stem Cell Transplantation University of Padova Padua Italy; ^13^ Pediatric Hematology and Oncology IRCCS Azienda Ospedaliero‐Universitaria of Bologna Bologna Italy; ^14^ Pediatric Department Scientific Institute for Research and Healthcare (IRCCS) San Gerardo dei Tintori Foundation Monza Italy; ^15^ Department of Hematology/Oncology and Cell and Gene Therapy Bambino Gesù Children's Hospital, IRCCS Rome Italy; ^16^ Department of Pediatrics Catholic University of the Sacred Hearth Rome Italy; ^17^ Department of Pathology and Laboratory Medicine Western University London Ontario Canada

**Keywords:** DBAS, episignature, methylation

## Abstract

Diamond‐Blackfan Anemia Syndrome (DBAS) is a rare inherited bone marrow failure syndrome (IBMFS) characterized by impaired erythropoiesis and significant genetic heterogeneity. Diagnosis can be challenging due to clinical variability and the lack of sensitive and specific biomarkers. We investigated the evidence for a DNA methylation (DNAm) episignature in a cohort of 80 DBAS patients with causative variants in various ribosomal protein genes: DBA1 (*RPS19*, *n* = 30), DBA4 (*RPS17*, *n* = 6), DBA5 (*RPL35A*, *n* = 8), DBA6 (*RPL5*, *n* = 15), DBA7 (*RPL11*, *n* = 13), DBA10 (*RPS26*, *n* = 8). We identified a distinct and highly accurate episignature biomarker for DBAS, clearly differentiating it from both Fanconi anemia and a broad spectrum of other episignature‐positive disorders. Furthermore, we developed a specific DNAm classifier for the clinically similar DBA6 and DBA7 subtypes. Applying the DBAS episignature analysis to six molecularly uncharacterized cases, three exhibited the DBAS pattern. Subsequent genome sequencing identified causative genetic variants in two (*RPL5*: c.325‐380A>G:p.?; *RPL26*: c.‐6 + 3_‐6 + 25del:p.?), validating the test robustness. Methylation profiles from two revertant cases (*RPS19*:P47L and *RPS17* full gene deletion) exhibited the DBAS episignature, suggesting it to be a stable epigenetic mark associated with the underlying genetic mutation, likely established early in development. In conclusion, we propose DNAm profiling as a robust diagnostic tool for DBAS, providing a biomarker applicable to all patients with clinical suspicion of the disease and critically aiding in the resolution of variants of uncertain significance and molecularly uncharacterized cases.

## Graphical abstract

Diamond‐Blackfan Anemia Syndrome (DBAS) is a congenital bone marrow failure disorder characterized by defective erythropoiesis and a spectrum of congenital anomalies, including craniofacial malformations, limb abnormalities, and cardiac and renal defects. DBAS is primarily caused by mutations in genes encoding proteins of the small and large ribosomal subunits. These genetic defects are associated with a distinct DNA methylation profile that differentiates individuals with DBAS from healthy controls. Notably, a specific episignature has been identified in patients belonging to the DBA6 and DBA7 molecular subgroups. DNA methylation profiling has proven useful for distinguishing patients with suspected DBAS but without known molecular alterations from those affected by other conditions that phenotypically resemble DBAS.

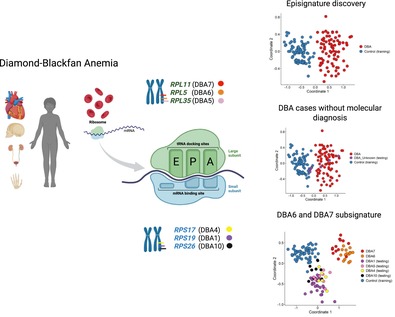

## 1 Introduction

Diamond Blackfan Anemia Syndrome (DBAS) is a rare, inherited bone marrow failure syndrome (IBMFS) characterized by macrocytic anemia, reticulocytopenia, and pure erythroid aplasia, typically presenting within the first year of life. This genetically heterogeneous condition also frequently involves non‐hematological manifestations, including physical malformations such as craniofacial anomalies, limb deformities, cardiac and renal defects, contributing to a complex and variable phenotype [1, 2, 3, 4, 5, 6]. Notably, a subset of individuals with DBAS‐associated genotypes may exhibit no overt hematological abnormalities, and diagnoses are increasingly made in adulthood with subtle presentations, highlighting the limitations of current diagnostic criteria [1, 2, 4, 7, 8, 9].

Initial steroids responsiveness is achieved in ~60%–80% of patients. However, only 30%–40% achieve a durable response, as many lose responsiveness or discontinue treatment due to side effects. The remaining patients become dependent on chronic red blood cell transfusions, with hematopoietic stem cell transplantation being the only curative option [1, 10, 11, 12, 13]. Notably, approximately 20% of patients achieve spontaneous, long‐term treatment independence [1, 2].

The genetic basis of DBA lies predominantly in heterozygous loss‐of‐function mutations in genes encoding ribosomal protein (*RP*) or their assembly factors, accounting for 70%–80% of cases. Mutations in *RPS19*, *RPL5*, *RPL11*, and *RPS26* are the most common [1, 14]. A smaller subset of cases is caused by mutations in non‐ribosomal genes, such as *GATA1* or gain‐of‐function *TP53* mutations (DBA‐other) [15, 16, 17, 18, 19].

This genetic heterogeneity and the variable penetrance of these mutations pose a significant diagnostic challenge. Unlike other IBMFS such as Fanconi anemia (FA), which has a definitive cellular assay (the DEB test), DBAS lacks a gold‐standard functional test. While biomarkers like elevated erythrocyte adenosine deaminase activity (eADA), and abnormal ribosomal RNA (rRNA) processing are supportive, they lack the specificity and sensitivity necessary for conclusive diagnosis [2, 20].

The diagnostic gap in DBAS highlights the urgent need for novel, robust biomarkers. DNA methylation (DNAm), a key epigenetic regulator of gene expression, exhibits stable and tissue‐specific patterns [21, 22]. Recently, several studies have identified characteristic DNAm signatures, or episignatures, associated with various genetic disorders, arising from pathogenic variants impacting epigenetic regulation [23]. Furthermore, a growing number of episignatures have been utilized as stable and reliable biomarkers for the diagnosis of congenital genetic disorders and for the reclassification of variants of uncertain significance (VUSs) [24, 25, 26, 27], and have been implemented in clinical diagnostic laboratories with significant diagnostic utility in genetically unresolved patients with suspected rare disorders [28, 29].

In this study, we identify and validate a novel and specific DNAm episignature for DBAS in peripheral blood. We demonstrate its potential as a robust diagnostic biomarker for distinguishing DBAS from other IBMFS and explore evidence for gene‐specific sub‐signatures. Finally, we analyze the episignature in patients who have achieved treatment independence via molecular reversion to elucidate the interplay between genotype, phenotype, and the stability of the epigenetic profile.

## 2 Methods

### 2.1 Subjects and Cohorts

The DBAS cohort comprised 86 individuals: 80 with a molecular diagnosis based on identification of likely pathogenic or pathogenic (LP/P) variants in known DBAS‐associated genes and six gene orphan subjects. The first cohort comprised 44 males and 36 females with a median age at diagnosis of 2 months (range: 0–209 months) and a median age at sampling of 51.5 months (range: 0–559 months). Congenital abnormalities were present in 54% (43/80) of patients, with craniofacial and upper limb anomalies being the most common. Steroid treatment was administered to 82.5% (66/80) of cases, resulting in partial or complete response in 57% (38/66), and steroid dependence in 29% (19/66). Six cases developed malignancies, and five patients died during follow‐up (Table 1).

**TABLE 1:** Features of DBAS patients.

	Overall	DBA1 (*RPS19*)	DBA4 (*RPS17*)	DBA5 (*RPL35A*)	DBA6 (*RPL5*)	DBA7 (*RPL11*)	DBA10 (*RPS26*)
Subjects (*n*)	80	30	6	8	15	13	8
Males	44	18	5	5	6	6	4
Females	36	12	1	3	9	7	4
Median, range age at diagnosis (months)	2 (0–209)	1 (0–29)	3.5 (2–13)	4.5 (0–54)	2 (0–209)	2 (0–50)	2.5 (0–11)
Median, range age at sampling (months)	51.5 (0–559)	21.5 (0–394)	339.5 (61–373)	29.5 (4–226)	90 (2–398)	72 (1–377)	13.5 (1–559)
Patients with congenital abnormalities	43	16	1	2	11	10	3
Type of congenital abnormalities							
Craniofacial	19	5	—	2	9	2	1
Upper limbs	17	3	—	—	5	9	—
Cardiovascular	10	3	—	1	—	4	2
Urogenital	9	5	1	—	1	1	1
Ophthalmological	4	2	—	—	1	—	1
Other	12	6	—	2	3	1	—
Steroid treatment							
Patients under treatment	66	26	5	6	11	12	6
Response to steroids (complete or partial)	38	13	3	2	10	7	3
Treatment status at last follows up							
Transfusion dependence	21	5	2	2	5	5	2
Steroid dependence	19	8	—	—	6	3	2
Spontaneous treatment independence	8	2	1	1	2	1	1
Treatment independence after steroids	6	3	3	—	—	—	—
Treatment independence after HSCT^a^	17	8	—	5	1	2	1
Lost at follow up	4	2	—	—	1	—	1
Malignancies	6	3	1	1	1	—	—
Status at last follow up							
Alive	75	28	6	8	15	11	7
Death	5	2	—	—	—	2	1

Abbreviation: HSCT, Human stem cell transplantation.

^a^ Methylation analysis was performed on DNA collected before HSCT.

An additional six patients with DBAS clinical diagnosis without identifiable causative variants in the primary DBAS genes (*RPS10, RPS17, RPS19, RPS24, RPS26, RPL5, RPL11, RPL35A*) were included and subsequently analyzed by genome sequencing.

The 80 patients with a confirmed molecular diagnosis were further classified into six subgroups based on the specific mutated *RP* gene. Notably, two patients (#25, DBA1; #35, DBA4) had achieved treatment independence due to documented molecular reversion. Detailed clinical features of the molecularly diagnosed cohort are summarized in Table 1 and Table S1, while clinical data for the six patients without a molecular diagnosis are presented in Table S2.

All patients underwent molecular analysis via sequencing of single genes, gene panels, or clinical exome sequencing (ES). Multiplex ligation‐dependent probe amplification (MLPA) analysis using the MRC‐Holland P212 assay was also performed for specific *RP* genes [30, 31].

The study included the following DBAS subgroups: DBA1 (OMIM #105650; *RPS19*, OMIM 603474; *n* = 30), DBA4 (OMIM #612527; *RPS17*, OMIM 180472; *n* = 6), DBA5 (OMIM #612528; *RPL35A*, OMIM 180468; *n* = 8), DBA6 (OMIM #612561; *RPL5*, OMIM 603634; *n* = 15), DBA7 (OMIM #612562; *RPL11*, OMIM 604175; *n* = 13), and DBA10 (OMIM #613309; *RPS26*, OMIM 603701; *n* = 8). The spectrum of LP/P mutations identified within each DBAS subgroup is detailed in Table S3. Variant classification followed the ACMG guidelines [32]; 47 variants were de novo, 24 unknown, and nine were inherited (Table S4).

All the samples and records were de‐identified prior to analysis. Written informed consent for the use of clinical information in this research was obtained from all patients by their treating physicians. This study was conducted under the auspices of the Multicenter Retrospective‐Prospective Observational Study on Diamond‐Blackfan Anemia, approved by the Ethics Committee of AOU Città della Salute e della Scienza di Torino, Turin, Italy (protocol number 0105777/2016), last amendment (0079681/2022) and the Western University Research Ethics Board (REB 106302).

### 2.2 DNA Methylation Analysis and Sample Processing

DNA methylation profiling was performed following previously described methodologies [26, 27]. Briefly, peripheral blood DNA was extracted using standard protocols, and methylation profiling was carried out using the Illumina Infinium MethylationEPIC BeadChip arrays (EPIC v.2) according to the manufacturer's instructions (Illumina, San Diego, CA, USA). IDAT files, containing methylated and unmethylated signal intensities, were imported into R (version 4.4.1). Normalization and probe‐level quality control (QC) were conducted using the SeSAMe package [33]. Probes were removed based on the following criteria: (i) probes located on sex chromosomes, (ii) cross‐reactivity with chromosomal locations other than their target regions, (iii) presence of SNPs at or near the tested CpG sites, and (iv) and probes with detection *p* value > 0.01. Principal component analysis (PCA) was conducted to assess potential batch effects and identify outlier samples.

Controls were randomly selected from EpiSign Knowledge Database (EKD; https://episign.lhsc.on.ca/index.html), at London Health Sciences Centre. Matching of controls and cases for age, sex, and array type was performed using the MatchIt package (version 4.5.1). Multiple matching trials were performed to optimize the sample size, with each analysis yielding a distinct number of matched controls. PCA was used to assess the structure of matched controls and confirm the absence of outliers in each trial.

Methylation levels (β‐values), ranging from 0 (no methylation) to 1 (full methylation), were logit‐transformed into M‐values. Differentially methylated probes (DMPs) were identified using linear regression modeling of M‐values, with false discovery rate (FDR) correction using the Benjamini‐Hochberg (BH) method (limma package). Blood cell proportions were estimated using the Houseman method [34] and included as covariates in the model. Each DBAS subgroup and the full DBAS cohort were compared to controls matched for age, sex, and array type to identify DMPs for each group. PCA was used to check the influence of common parameters such as age, sex, and array type on data structure, and none was observed. Detailed information for each patient was not available for steroid therapy and this was not included as a covariate in the model.

Next, the mean methylation differences between cases and controls were multiplied by the negative log‐transformed adjusted *p* value, and the top 900 probes with the highest scores were selected for further analysis. Receiver operating characteristic (ROC) analysis was then performed to identify the 450 probes with the highest area under the curve (AUC). Probes with Pearson's correlation coefficients > 0.65 between cases and controls were excluded. The final set of 206 probes remained for further analyses. Hierarchical clustering (Ward's method, Euclidean distance) and multidimensional scaling (MDS) were performed using the gplots package in R.

A machine learning support vector machine (SVM) model was developed using the e1071 R package to generate a Methylation Variant Pathogenicity (MVP) score, applying Platt's method [35] to transform the raw SVM output into a probability indicating the likelihood that a sample is associated with the episignature under examination. MVP scores closer to 1 indicate a high probability that the methylation pattern corresponds to the condition being studied. The model was trained using target cases, matched controls, 75% of other controls, and 75% of subjects with other rare genetic disorders from the EKD. The remaining 25% of other controls and 25% of subjects with other disorders were used as the testing set for the SVM classifier. This procedure was employed to assess the performance of the classifier on an independent testing set, ensuring a more robust evaluation of the model's predictive ability.

Multiple rounds of leave‐one‐out cross‐validation (LOOCV) were conducted in each analysis to evaluate the reproducibility and robustness of the episignatures. In each round, one case was withheld as the test set, while the remaining cases were used for probe selection and model development. Probe selection was performed independently in every iteration, followed by MDS plotting, heatmap generation, and SVM classification using the corresponding probe set. Due to the large number of samples and multiple cohorts, only the summary of the MVP scores is reported. Additional unsupervised clustering analyses yielded results consistent with the MVP summary.

### 2.3 Detection of Differentially Methylated Regions and Gene Ontology Analysis

Differentially methylated regions (DMR) were detected using the DMRcate package (version 2.12.0). A genomic region was considered a DMR if encompassing at least five different CpG sites within 1 kb with a mean methylation difference of 10% between case and control and a Fisher's multiple comparison *p* value of < 0.01.

The missMethyl package (version 1.38.0) was used to perform Gene Ontology (GO) analysis on the identified DMRs. The DMRs were assessed for GO term enrichment, accounting for variations in the number of probes per gene on the array and CpGs mapped to multiple genes. The analysis assumed an equal likelihood for all genes to be associated with significant CpG sites, and the FDR was calculated using the BH method.

### 2.4 Cohort Similarity and Functional Correlation Analysis

Similarity between cohorts and functional correlation were assessed as previously described by Levy et al. [36]. Each DBAS subgroup, as well as the entire DBAS cohort, was compared to age‐, sex‐, and array‐matched controls to identify DMPs in each group. Controls were selected to exclude individuals with rare genetic disorders or known episignatures. Probes were considered differentially methylated if the mean methylation difference between cases and controls was greater than 5%, with a BH‐corrected *p* < 0.01.

DMPs shared between the DBAS cohorts and 99 other disorders from the EpiSign Knowledge Database (EKD) were visualized using a heatmap (heatmap R package, version 1.0.12). A tree‐and‐leaf diagram was generated by ranking DMPs based on *p* values, with the top 500 DMPs from each cohort selected to evaluate the relationship between DBAS and the 99 other EKD disorders. Euclidean clustering (Ward's method) was applied to the matrix, and a tree‐and‐leaf plot was generated using the TreeAndLeaf R package.

DMPs were annotated relative to CpG islands (CGIs) and genes using the AnnotationHub R package (version 3.12.0) as the annotation resource, incorporating hg19_cpgs, hg19_basicgenes, hg19_genes_intergenic, and hg19_genes_intronexonboundaries. CGI annotations included CGIs, CGI shores (0–2 kb flanking CGIs), CGI shelves (2–4 kb flanking CGIs), and inter‐CGI regions. Gene annotations included promoters, promoter+, and gene bodies. Promoters were defined as the genomic region within 1 kb upstream of the transcription start site, promoter+ covered regions 1–5 kb upstream of the transcription start site, and the “gene body” category encompassed untranslated regions, exons, introns, and exon/intron boundaries.

### 2.5 Genome Sequencing Analysis

The library was prepared using the library preparation kit TruSeq DNA Nano (Illumina) using the NovaSeq X Plus, 2 × 151 bp as sequencing parameters. Demultiplexing of the sequencing reads was performed with Illumina bcl2fastq (version 2.20). If more output was generated for a sample than requested, the reads of this sample were downsampled to at least 20% above the ordered output. Adapters were trimmed with Skewer (version 0.2.2) [37]. Quality trimming of the reads has not been performed. The MultiQC report was generated using MultiQC version 1.22.2 (https://multiqc.info/). The quality of the FASTQ files was analyzed using FastQC on Illumina's DRAGEN Bio‐IT Platform (version 4.2.4). Plots were created using ggplot2 [38] in R (version 4.0.4) (R Core Team 2015—available online at https://www.R‐project.org/). After sequencing, the FASTQ files were processed with in‐house pipelines managed by Nextflow in a secure cloud computing infrastructure available at Computational Genomic at IRCCS Azienda Ospedaliero‐Universitaria di Bologna, optimized for efficient processing of genomic data on GPU‐ accelerated systems. In particular, for germline variant analysis, we used a GPU‐accelerated version of NVIDIA Clara Parabricks to improve performance for alignment and calling. The raw reads were aligned to the human reference genome GRCh38, which contains the primary assembly as well as ALT contigs, additional decoy contigs and HLA genes. Alignment and calling of small variants was performed with the Germline Pipeline of NVIDIA Clara Parabricks (v4.2.1), using BWA as aligner and DeepVariant as caller. The variants were annotated with VEP (v.108). Each variant was annotated with genes, transcripts and sequences and other useful predicted values derived from CADD (v.1.6), polyphen (v.2.2.2), SIFT (v.5.2.2), MPC (from dbNSFP4.3a_grch38) and allele frequency information from GnomAD genome allele frequency (v.4), predictions of splicing influences and UTR regions with SpliceAI and MaxEntScan and UTRannotator, clinical information such as ClinVar annotations (202205) and for non‐coding annotations different scores such as Gnocchi, ncER and ReMM were used. The structural variants (SVs) were obtained using an in‐house pipeline that combined the variants discovered by various tools such as Manta, Delly and Smoove and merged them with SURVIVOR. SVs were annotated using VEP (v.108) with the specific plugin and allele frequency information was obtained from gnomADSV (v.4). Different types of Bening datasets were used for each type of SVs, such as ClinVar, ClinGen, DGV, dbVar and 1000 Genomes.

### 2.6 Minigene Splicing Assay

To validate the computational predictions and investigate the RNA splicing profile associated with the *RPL5* c.325‐380A>G variant in individual #82, we performed an in vitro splicing assay using the pSPL3 vector producing a construct including the entire *RPL5* exon 5 (203 bp) flanked by part of the upstream intron (472 bp), containing the variant nucleotide, and of the downstream intron (144 bp). HEK293T cells were transfected with either the wild‐type (c.325‐380A) or variant (c.325‐380G) constructs, followed by RNA extraction 48 h post‐transfection. The pSPL3 vector model includes two exons, a splice donor (SD), splice acceptor (SA), a functional intron, SV40 promoter, and late poly A signal (LPAS). The forward primer SD6‐5′‐TCTGAGTCACCTGGACAACC and reverse primer SA25′‐ATCTCAGTGGTATTTGTGAGC were used for PCR amplification. The empty vector produces a 263 bp cDNA fragment. The anticipated amplicon size for the wild‐type isoform was 466 bp, representing 203 bp of exon 5 from the *RPL5* gene.

## 3 Results

### 3.1 A Distinct DNA Methylation Episignature in DBAS Patients

**FIGURE 1:**
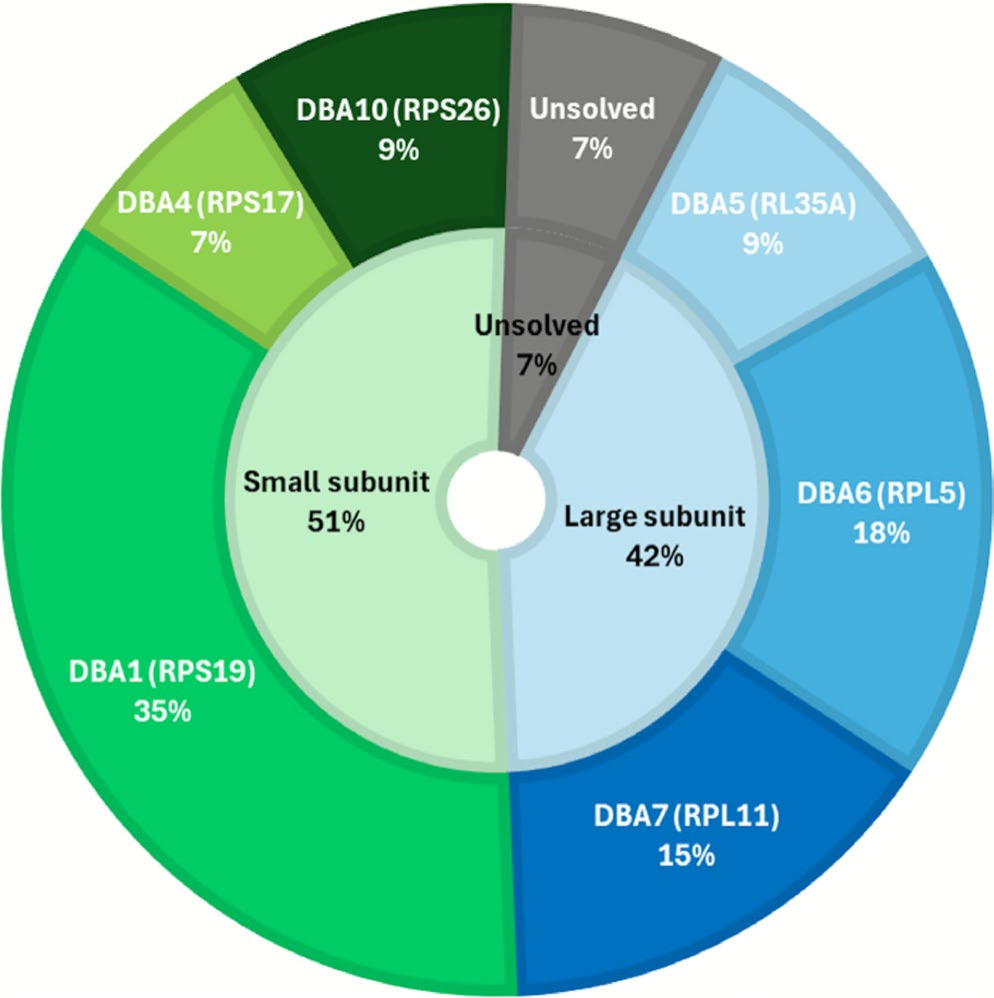
Genetic characterization of the entire DBAS patient cohort. Representation of DBAS subgroups associated with mutations in minor ribosomal subunit genes, those associated with alterations in major ribosomal subunit genes, and the subgroup of patients with suspected DBAS but unknown molecular alterations. [Color figure can be viewed at wileyonlinelibrary.com]

**FIGURE 2:**
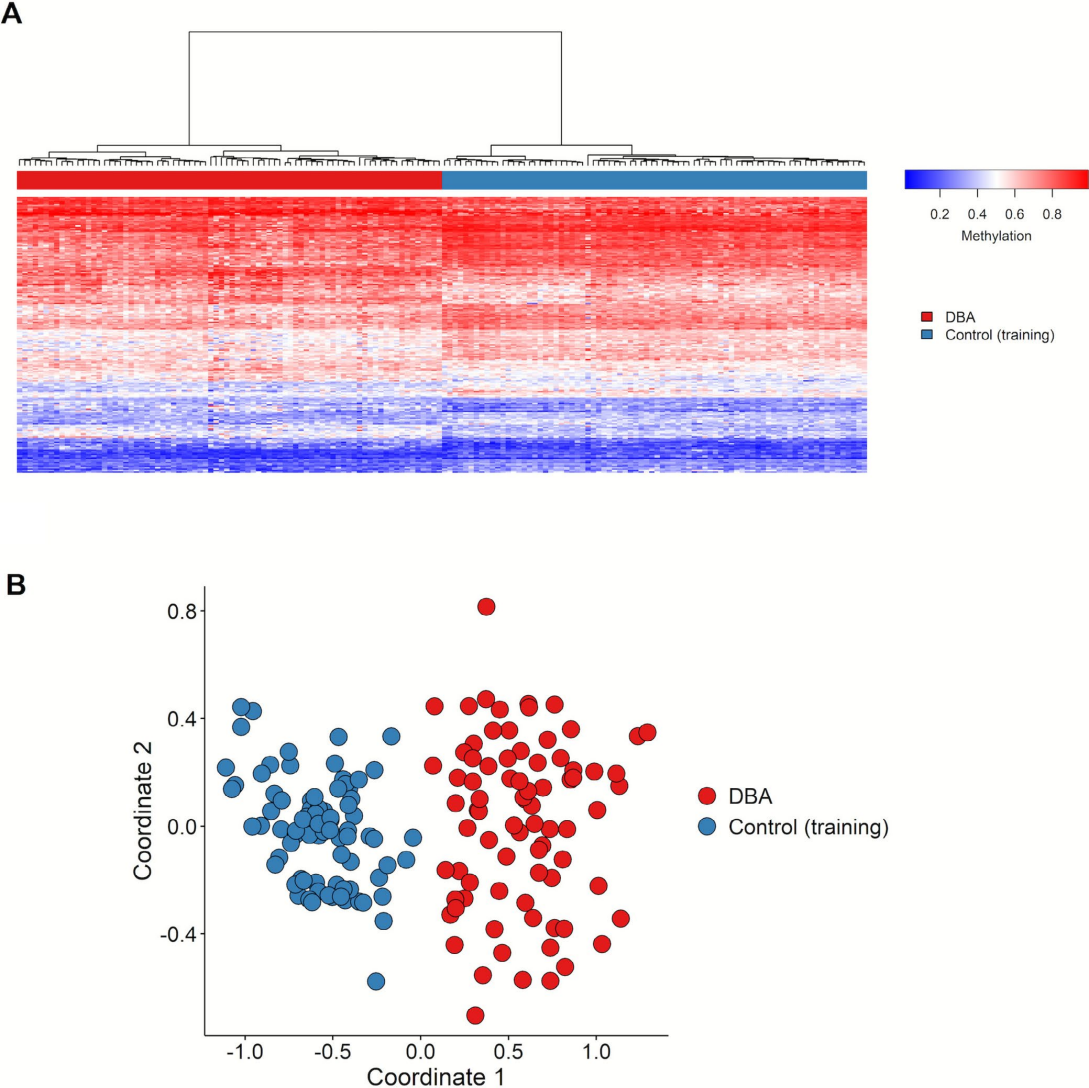
Discovery of an episignature in DBAS cohort. (A) Heatmap of DNA methylation patterns: each column is one individual (either with DBAS or a healthy control). Each row is one of the 206 differentially methylated CpG probes identified by comparing the DBAS cases versus controls. The red columns are DBAS cases, and the blue columns are controls. A clear separation was observed between DBAS cases and controls. (B) Plot of samples similarity: the multidimensional scaling (MDS) plot shows how the DBAS cases (red) cluster apart from the controls (blue) based on their DNA methylation patterns. [Color figure can be viewed at wileyonlinelibrary.com]

We performed DNAm analysis on 80 patients with LP/P variants in *RPS17*, *RPS19*, *RPS26, RPL5*, *RPL11*, *RPL35A* (Figure 1). Comparing DBAS cases with matched controls from the EpiSign Knowledge Database resulted in the selection of a total of 206 DMPs that define a specific DBAS episignature (Supporting Information, Data 1). Heatmap and MDS plots demonstrated a clear separation between DBAS cases and controls (Figure 2A,B). Training cases exhibited low MVP scores near 0 for other disorders, indicating high specificity of the developed model (Figure S1A). LOOCV analysis further supported a robust DBAS episignature, with most testing samples exhibiting high MVP scores (Figure S1B) and clustering with training cases in MDS plots (Figure S2). Overall sensitivity, for cases with a confirmed DBAS mutation was 1.00, and specificity was close to ~1.00.

### 3.2 Specificity of the DBAS Episignature Relative to FA

**FIGURE 3:**
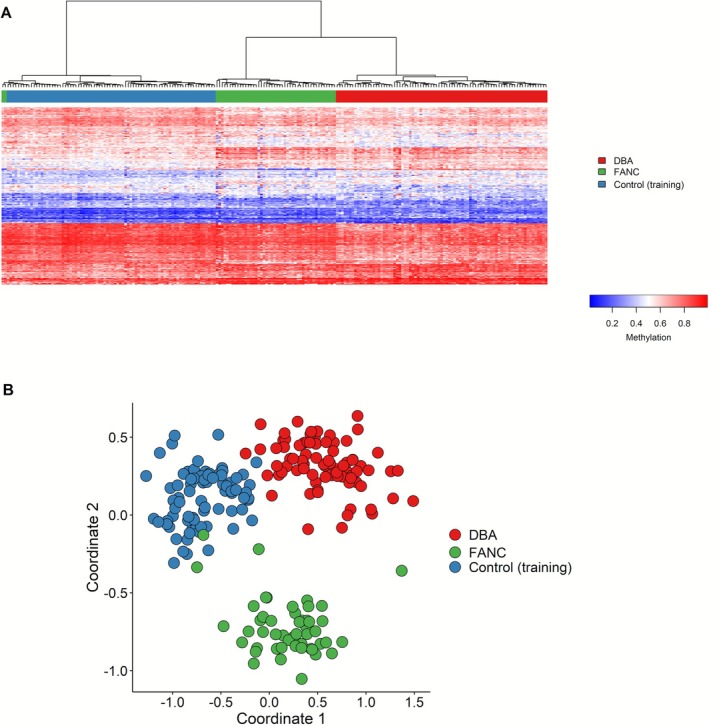
DNA methylation profiling of DBAS, FA (FANC), and control samples. (A) Heatmap showing the separation of methylation profiles between DBAS cases (red), FA cases (green), and control samples (blue). (B) MDS plot illustrating the distinct clustering of DBAS cases (red), FA cases (green), and control populations (blue) based on their methylation profiles. [Color figure can be viewed at wileyonlinelibrary.com]

Given the clinical overlap between DBAS and FA, we tested the DBAS episignature against a cohort of FA patients using both supervised and unsupervised clustering. Heatmap and MDS plots clearly distinguished FA cases from DBAS cases, highlighting underlying epigenetic differences despite similar clinical presentations including a common anemia phenotype (Figure 3). Furthermore, FA cases consistently exhibited MVP scores close to 0 in the MVP plot, further emphasizing the specificity of the DBAS episignature for DBAS compared to FA (Figure S1A).

### 3.3 DNA Methylation Profiling in Different Subgroups of DBAS

To investigate DNAm patterns within specific DBAS subtypes, the previously described methodology was applied to develop six independent, subtype‐specific classifiers. Models were trained separately for DBA1 (*n* = 30), DBA4 (*n* = 6), DBA5 (*n* = 8), DBA6 (*n* = 15), DBA7 (*n* = 13), and DBA10 (*n* = 8), utilizing the remaining subtypes and controls as the testing set. Heatmap and MDS plots consistently showed a clear separation between cases and controls for each analyzed subtype. MVP scores were close to 1 for training cases, while controls and cases from the external validation cohort (EKD) exhibited MVP scores near 0 (Figures S3–S8). LOOCV test confirmed highly reproducible results in larger cohorts (DBA1, DBA6, and DBA7). However, smaller subtypes (DBA4, DBA5, and DBA10) showed a higher proportion of cases with lower MVP scores (< 0.25).

**FIGURE 4:**
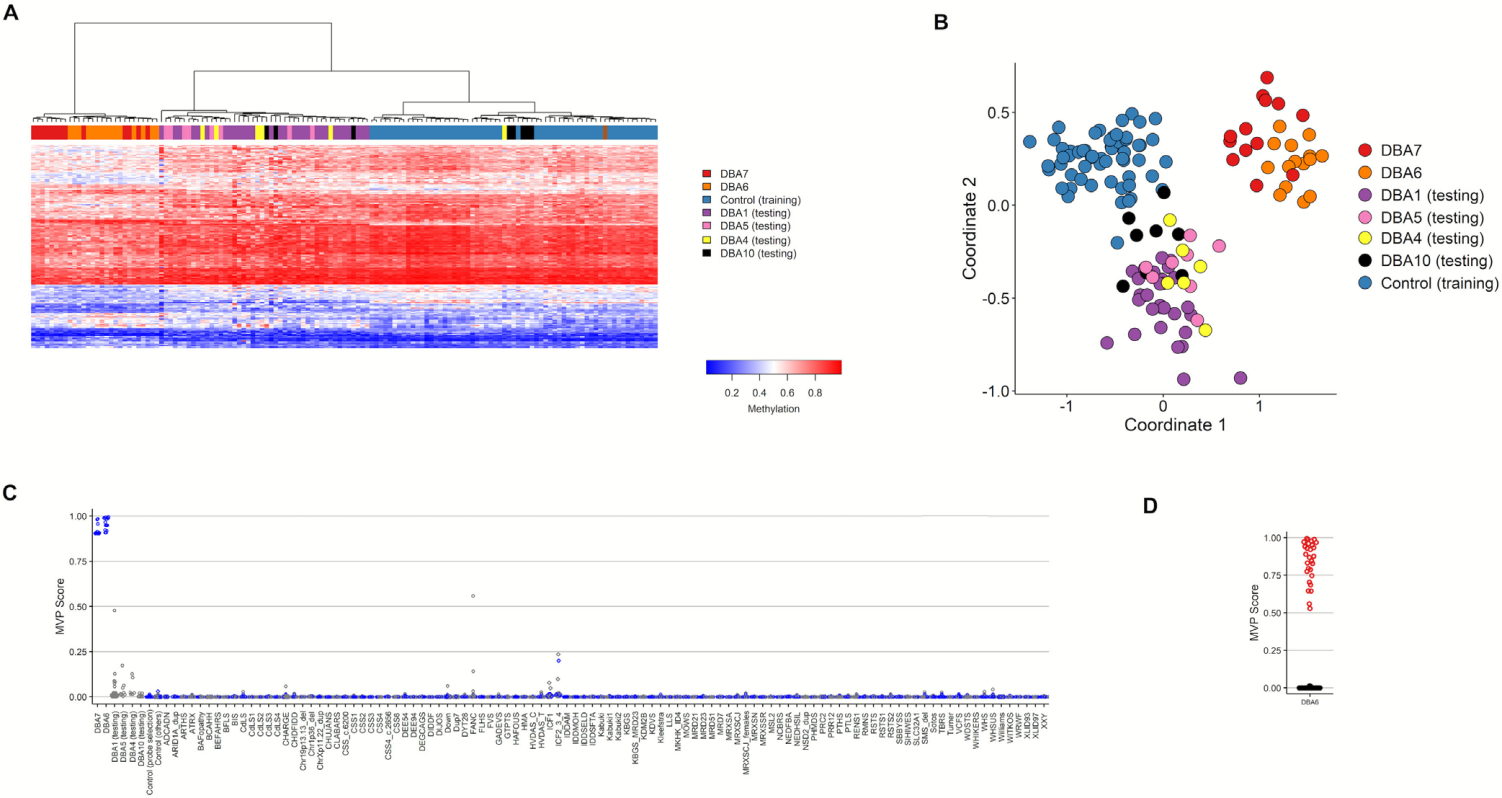
Discovery of a sub episignature for DBA6 and DBA7 cohorts. (A) Heatmap that shows a shared DNA methylation pattern in DBA6 (orange) and DBA7 (red) samples clearly distinct from the controls (blue) and other DBAS subtypes (purple = DBA1, yellow = DBA4, pink = DBA5, black = DBA10). (B) MDS plots that demonstrated the presence of a distinct cluster corresponding to DBA6 (orange) and DBA7 (red) differentiating them from controls (blue) and other DBAS cases (purple, yellow, pink, and black). (C) SVM classifier, a type of supervised machine learning algorithm, built to distinguish between DBA6/DBA7 cases and other samples. The algorithm was developed in two steps. First, during the *training* phase, it learned to recognize the pattern of DBA6/DBA7 cases (*n* = 28) compared with their matched controls, two‐thirds of other controls, and other disorders with detectable DNA methylation patterns (shown as blue circles). In the second, *testing* phase, the remaining samples (one‐third of controls and other disorders) were used to evaluate the model's performance (gray circles). Additional DBAS subtypes (DBA1, DBA4, DBA5, DBA10) were also tested (gray circles). The model assigned scores close to 1 for DBA6/DBA7 samples and near 0 for other DBAS subtypes and unrelated disorders, indicating the specificity of the model in detecting the methylation signature of DBA6/DBA7. (D) MVP score, a metric derived from the SVM classifier, was calculated for each DBA6 and DBA7 case using a leave‐one‐out approach (each case tested individually while the others trained the model) and compared with the average scores of controls and other episignature disorders across all rounds. DBA6/DBA7 cases (red dots) consistently scored high (near 1), while controls and other disorders stayed low (near 0), confirming the distinct and reproducible episignature. The abbreviations for the disorders are listed in Table S5. [Color figure can be viewed at wileyonlinelibrary.com]

Notably, independent analysis of DBA6 or DBA7 revealed a distinct shared cluster encompassing cases from both subtypes, separate from other subtypes and controls (Figure S9). Based on this observation, DBA6 and DBA7 were combined to develop a unified model. This combined model yielded a highly specific classifier for these two subtypes (Figure 4). Heatmap and MDS plots showed that DBA6 and DBA7 cases clustered together, clearly separated from other subtypes which grouped closer to controls. The high MVP scores observed in DBA6 and DBA7 cases, compared to lower scores (< 0.50) in other subtypes, further supported the specificity and sensitivity of this combined model. All testing cases in the LOOCV of this unified model also yielded high MVP scores (> 0.50), confirming its reproducibility.

### 3.4 Application of DNA Methylation Profiling in DBAS Cases Without Prior Molecular Diagnosis

**FIGURE 5:**
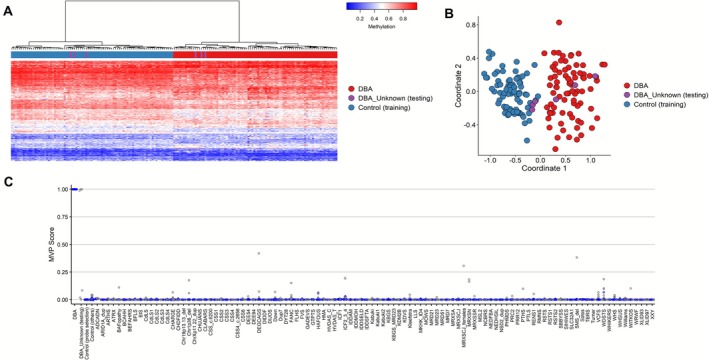
Testing the methylation profile of DBAS cases without molecular diagnosis. (A) Heatmap: Each column represents an individual DBAS case (red), control (blue), or unsolved DBAS case (purple). (B) MDS plot showing the clustering of DBAS cases (red), unsolved DBAS molecular cases (purple), and controls (blue). (C) SVM classifier: the model highlights three unsolved DBAS samples with high MVP scores (near 1), while the remaining three unsolved DBAS samples have MVP scores below 0.25 (gray circles). The abbreviations for the listed epigenetic profiles are provided in Table S5. [Color figure can be viewed at wileyonlinelibrary.com]

Individuals with clinical features suggestive of DBAS but lacking molecular findings were then tested using the established pan‐DBAS classifier (Figure 1). Three out of six cases (cases #81, #82 and #83) clustered with molecularly confirmed DBAS cases in both heatmap and MDS plots (Figure 5A,B) and exhibited high MVP scores for the DBAS episignature (Figure 5C and Table S2).

Genome sequencing was performed on all six individuals without a prior molecular diagnosis.

In patient #82, we identified a novel deep intronic variant in *RPL5* (c.325‐380A>G:p.?). This variant was not present in the gnomAD (v.4.1.0) database. Computational analysis using SpliceAI predicted the creation of a new acceptor splice site at position +1 relative to the mutation (score: 0.85) and a novel donor splice site at position +79 (score: 0.83). These changes were anticipated to lead to the retention of a 79‐bp intronic segment within the *RPL5* transcript. Functional validation through minigene splicing assays demonstrated that the mutant (c.325‐380G) transcript, unlike the wild‐type (c.325‐380A), indeed retained the predicted 79‐bp intronic sequence (Figure S10A–C). The retention of this sequence is predicted to induce a frameshift mutation, resulting in the alteration of three amino acids and the introduction of a premature stop codon at position 112 (Figure S10D). Clinical manifestations in this patient included typical somatic malformations, steroid dependency, elevated eADA levels, and a rRNA profile on Bioanalyzer consistent with DBA6/DBA7 patients (Table S2, Figure 5).

In individual #83, a likely pathogenic variant, *RPL26*:c.‐6 + 3_‐6 + 25del:p.?, was detected. Splicing predictions indicated a high likelihood of pathogenicity (SpliceAI = 0.96, Pangolin = 0.67). This variant has previously been reported in a family with various DBAS‐related clinical features [39]. Functional analysis has further confirmed its pathogenicity. This patient presented with a thumb malformation, frequently described in patients with *RPL26* variants [8, 39], elevated eADA levels, and a Bioanalyzer rRNA profile consistent with Northern blotting results from a patient with an *RPL26* mutation [40] (Table S2, Figure 5).

Individual #81 was genetically unresolved by genome sequencing but had a clinical diagnosis of DBAS based on neonatal anemia and markedly elevated eADA levels. The disease was severe enough to require chronic transfusions and eventual HSCT. An rRNA analysis for this patient was not possible due to poor RNA quality.

In contrast, patients #84, #85, and #86 also had elevated eADA, but their rRNA ratios were within the normal range [2, 20]. These three cases are currently treatment‐independent, though they maintain a mild hematological phenotype.

### 3.5 DNA Methylation Profile in Reverted DBAS Patients

**FIGURE 6:**
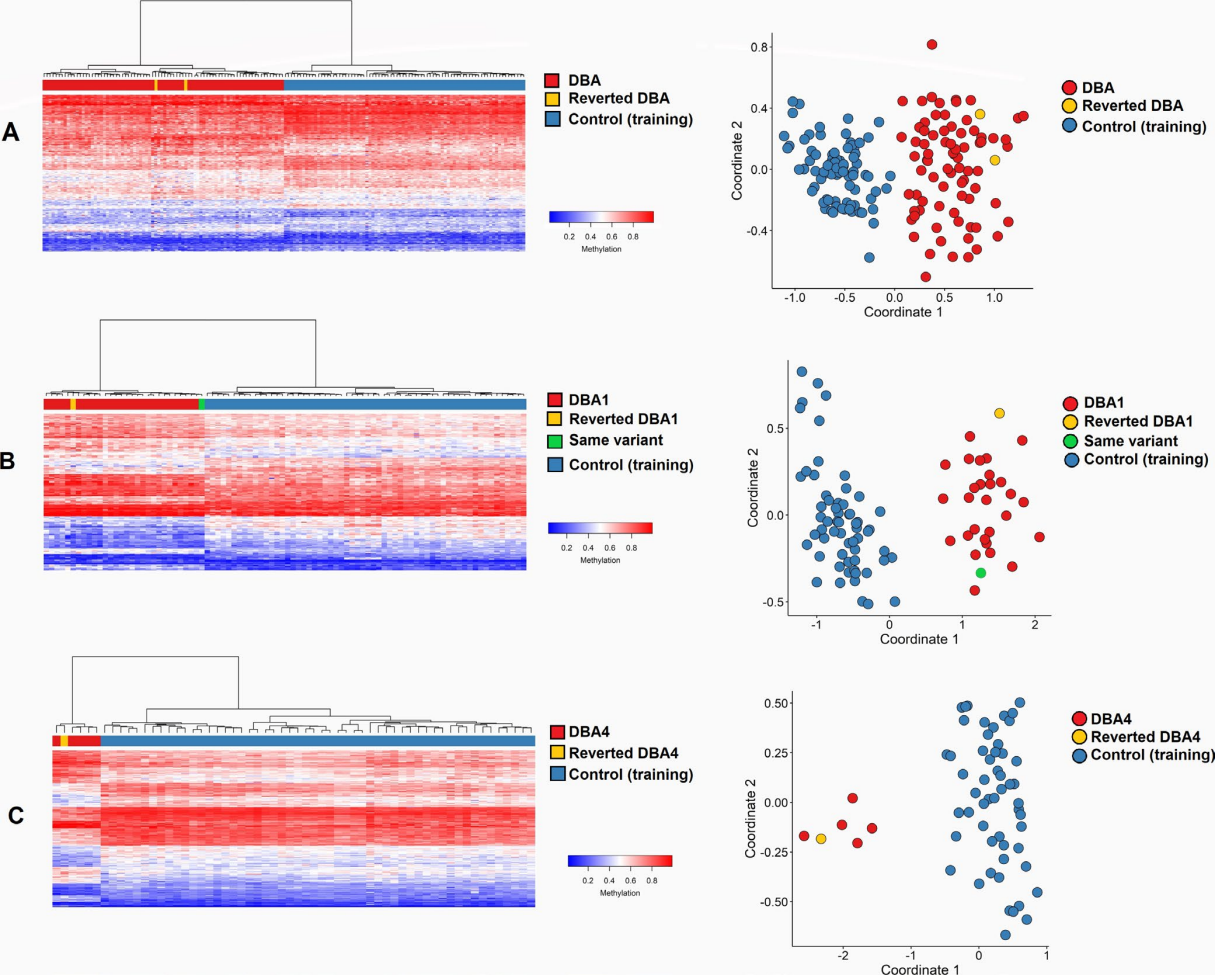
Testing the DBAS cases with molecular reversion. Heatmaps (left) and MDS plots (right) illustrate the DNA methylation profiles of (A) the entire DBAS cohort, (B) the DBA1 subtype, and (C) the DBA4 subtype. In each analysis, cases with molecular reversion (yellow) were used as the testing set while other DBAS cases (red) and matched controls (blue) were used to train the model. No differences were observed between the reverted case patient #25 (yellow) and patient #26 (green), which carries the same variant, in the DBA1 cohort. Similarly, patient #35 (yellow) clustered alongside the training DBA4 cases (red), indicating no significant deviation in methylation patterns. [Color figure can be viewed at wileyonlinelibrary.com]

The DBAS cohort included two patients (#25 and #35) who had achieved treatment independence (reverted DBAS). Both cases displayed a methylation profile consistent with affected individuals who are non‐molecular revertants (Figure 6). Patient #25 harbored a missense mutation in the *RPS19* gene (p.Pro47Leu). This proband presented with severe macrocytic hyporegenerative anemia at birth, hypoplasia of the right thenar eminence, and elevated eADA activity, leading to a clinical diagnosis of DBAS. The patient was steroid‐unresponsive and regularly transfused. At 8 years of age, stable hematological treatment independence was achieved and maintained at the last follow‐up. High‐density SNP arrays identified a mosaic segmental 29.7 Mb loss of heterozygosity on chromosome 19q12–q13.43, indicative of multiple clones with uniparental disomy (UPD) [41]. Interestingly, patient #26, a non‐revertant with the same p.P47L missense mutation, showed a similar DNAm profile (Figure 6B).

Patient #35 achieved treatment independence with normalization of eADA value after a long period of steroid therapy. This was attributed to revertant UPD ablating a complete *RPS17* deletion detected by high‐density SNP arrays (Figure 6C).

### 3.6 Functional Correlation and Comparison With Other Disorders

To assess the specificity of the DBAS episignature the entire DBAS cohort subjects and individual DBAS subtypes were compared to other episignature positive disorders from the EKD (a complete list of EpiSign v5 disorders and their abbreviations is provided in Table S5). A total of 5541, 9728, 24 065, 10 208, 9343, 5790, and 2514 DMPs were identified for the entire DBAS cohort, DBA1, DBA4, DBA5, DBA6, DBA7, and DBA10, respectively. The highest overlap of genome‐wide DMPs (45%) was observed between the entire DBAS cohort and DBA1, which represented the larger sample size of the DBAS cohort. Notably, DBA6 exhibited its highest probe overlap with DBA7 (39%), suggesting closer epigenetic similarity between these two subtypes. Additionally, DBA1 shared 45% of its probes with DBA10, while DBA4 showed the highest probe overlap with DBA5 (Figure S11). Among other disorders in the EKD, Mowat‐Wilson syndrome (MOWS) (25% DMP overlap), velocardiofacial syndrome (VCFS) (23%) and Neurodevelopmental disorder with hypotonia, stereotypic hand movements, and impaired language (NEDHSIL) (22%) had the highest DMPs overlapped with the entire DBAS cohort.

The mean methylation differences were also compared between DBAS and other disorders, revealing an overall hypermethylation trend in the entire DBAS cohort (Figure S12). While DBA4, DBA6, and DBA7 showed a predominant hypomethylation pattern, DBA1, DBA5, and DBA10 revealed general hypermethylation trends. The relationships between cohorts were analyzed using the top 500 most significant probes from each group. In the tree and leaf plot (Figure S13), all individual DBAS cohorts clustered closely together. Notably, the hypomethylated cohorts (DBA4, DBA6, and DBA7) formed a distinct cluster, while the predominantly hypermethylated cohorts (DBA1 and DBA10) clustered within the same branch. Interestingly, certain blood‐affecting syndromes, such as FA, and DEGCAGS syndrome (Developmental delay with gastrointestinal, cardiovascular, genitourinary, and skeletal abnormalities syndrome), clustered near the main DBAS cohorts.

Considering the entire DBAS cohort, annotation analysis showed that DMPs were predominantly located in inter‐CGI regions (46%) and CpG islands (29%) (Figure S14A). Gene annotation revealed that the majority of DMPs were mapped to coding regions (~40%) and intergenic regions (~24%) (Figure S14B). In the GO enrichment analysis of the whole cohort, only the molecular function term “peptide antigen binding” was found to be significant. However, 3, 44, 7, 43, and 43 significant terms (FDR < 0.05) were identified for DBA1, DBA4, DBA5, DBA6, and DBA7, respectively. A subsignature including DBA6, and DBA7 was associated with 11 GO terms. No significant GO terms were detected in the DBA10 analysis (Supporting Information, Data 2).

### 3.7 Differentially Methylated Regions

A total of three DMRs were identified across all DBAS samples, including two hypermethylated and one hypomethylated region, all overlapping with gene regions (Supporting Information, Data 3). Similarly, the number of identified DMRs for each DBAS subtype varied: DBA1 (15), DBA4 (14), DBA5 (36), DBA6 (16), DBA7 (4), and DBA10 (8). A frequently observed DMR on chromosome 19 (50,860,847‐50,862,121) including NR1H2 and NAPSA genes was present across all subtypes except DBA5. Additionally, a DMR on chromosome 6 (106,545,984‐106,546,824) including PRDM1 was consistently detected in DBA1, DBA4, DBA5, and the entire DBAS cohort.

## 4 Discussion

This study establishes a unique and robust DNAm episignature specific to DBAS, clearly distinguishing patients from healthy controls and those with other relevant genetic disorders, notably FA. Our findings highlight the significant potential of DNAm profiling as a diagnostic tool for DBAS, which is particularly valuable given the genetic heterogeneity, variable clinical presentation, and expanding list of causative genes that complicate diagnosis [1]. The clinical utility of this approach was underscored by its successful application to molecularly unresolved cases. The pan‐DBAS classifier prioritized individuals for further genetic investigation, leading to the identification of causative variants in two out of three positive cases: a deep intronic *RPL5* variant (c.325‐380A>G) in patient #82, whose clinical features aligned with DBA6, and a known pathogenic *RPL26* splice site deletion (c.‐6 + 3_‐6 + 25del) [39] in patient #83. This demonstrates the utility of episignature analysis to guide genetic diagnosis even when the specific subtype signature is unavailable, or the presentation is atypical. Conversely, the absence of the episignature in three other unconfirmed cases suggests they may have conditions that phenotypically mimic DBAS, reinforcing the episignature specificity for the canonical disease.

Beyond the general DBAS signature, we identified a distinct, predominantly hypomethylated episignature for the combined DBA6/DBA7 (*RPL5*/*RPL11*) cohort. While not resolving into gene‐specific patterns, this shared signature links two genes on chromosome 1p with a higher incidence of somatic malformations and elevated eADA [2, 42, 43]. The underlying mechanisms may relate to the critical roles of *RPL5* and *RPL11* in p53 regulation via MDM2 inhibition, as p53 pathways influence epigenetics and are frequently activated by ribosomal stress [44]. Within this subtype, we also identified hypermethylation of *SPRED3*, a negative regulator of Ras/MAPK signaling. Given that this pathway is critical for hematopoietic cell proliferation and differentiation, its epigenetic dysregulation could contribute to DBAS pathophysiology [45]. Additional subsignatures for other genetic subtypes were not identified, likely due to small sample sizes, but could emerge as larger patient cohorts are analyzed.

Intriguingly, the DBAS episignature persisted in two cases (#25 and #35) who achieved clinical treatment independence through somatic reversion (mosaicism/UPD leading to loss of the germline mutation), a finding that mirrors observations in FA [29]. Despite their complete hematological recovery, their methylation profiles remained indistinguishable from non‐revertant DBAS patients. This critical finding establishes the episignature as a stable developmental imprint of the underlying genetic defect, rather than a dynamic marker of disease activity. Analysis of differentially methylated regions in the corresponding subtypes (DBA1, DBA4) highlighted genes involved in inflammatory responses, immune regulation (*ELANE, NR1H2*, and *PRDM1*) and carcinogenesis (*ELANE, KLK4*, *PRDM16*, and *PRSS21*). For instance, alterations in *PRDM16* are directly linked to MDS/AML development while *ELANE* and *KLK4* are known to be pro‐tumorigenic [46, 47, 48]. The persistent epigenetic alterations in these genes might reflect the systemic impact of ribosomal dysfunction and could contribute to long‐term clinical risks, particularly the increased malignancy risk characteristic of DBAS.

Genome‐wide, methylation changes in DBAS overlapped significantly with those of Mowat‐Wilson syndrome (*ZEB2*) and Velocardiofacial syndrome (VCFS; *TBX1*, *DGCR8*), suggesting shared downstream developmental pathways affected by ribosomal stress. The connection to *ZEB2*, a transcription factor involved in epithelial–mesenchymal transition and development, is interesting given its role in pathways potentially influenced by ribosomal stress and p53 [49, 50]. The overlap with VCFS is supported by previous findings of *TBX1* downregulation in *RPS19*‐deficient models [51], suggesting molecular links between ribosomal function and developmental pathways affected in VCFS. Furthermore, clustering analysis placed all DBAS subtypes together, grouping closely with other inherited bone marrow failure syndromes like FA and DEGCAGS syndrome.

Annotation of differentially methylated positions across the DBAS cohort showed enrichment in inter‐CGI regions and CpG islands, mapping primarily to coding and intergenic regions.

While pathway enrichment for the entire cohort was limited, subtype‐specific analyses revealed numerous significant terms, indicating potential functional divergence in the epigenetic impact across different genetic forms of DBAS.

The primary limitation of this study is the small sample size for some subtype‐specific analyses (e.g., DBA4, DBA5, DBA10), which likely affected their classification accuracy (e.g., #34‐*RPS17*, #44‐*RPL35A*). Expanding cohort sizes through international collaboration is essential to refine these subtype‐specific signatures.

In conclusion, this work establishes DNA methylation profiling as a robust diagnostic biomarker for patients with suspected DBAS. A key strength is its stability, persisting even after the state of treatment independence following molecular reversion, making it a reliable indicator of the underlying genetic defect.

This approach is poised to supersede traditional methods like Northern blot and rRNA profiling, which are technically challenging and less specific. Crucially, it also offers a powerful tool for resolving VUS—a major diagnostic hurdle.

Future work should integrate these epigenetic data with clinical and molecular findings to unravel phenotypic variability in DBAS and expand this diagnostic framework to rarer subtypes, phenocopies, and related ribosomopathies like Shwachman‐Diamond syndrome, Treacher Collins syndrome, and congenital dyskeratosis.

## Author Contributions

P.Q., E.G., S.T., K.K., A.B., B.S.: conceptualization. P.Q., M.Z., F.F., A.B., S.C., M.G., M.L., M.E.C., G.Z., F.T., P.C., U.R., F.F.: patients' recruitment. K.K., S.T., E.G., M.S., G.P., S.D., L.C., E.I., T.P., J.K., J.R., H.M.C., M.L., S.R., A.C., B.S.: data collection and analysis. ABr, BS, ST, KK, PQ, EG: supervision. PQ, KK, EG, ST, ABr: writing – original draft. All authors: writing – review and editing.

## Ethics Statement

This study was conducted under the auspices of the Multicenter Retrospective‐Prospective Observational Study on Diamond‐Blackfan Anemia, approved by the Ethics Committee of AOU Città della Salute e della Scienza di Torino, Turin, Italy (protocol number 0105777/2016), last amendment (0079681/2022) and the Western University Research Ethics Board (REB 106302). The study was conducted in accordance with the Declaration of Helsinki and relevant national regulations.

## Conflicts of Interest

B.S. is a shareholder in EpiSign Inc. All other authors declare no conflicts of interest.

## Supporting information


**Appendix S1:** Supporting Information.

## Data Availability

The data that support the findings of this study are available on request from the corresponding author.
