# Supplementary material for: DNA Methylation Episignature as a Novel Diagnostic Tool for Diamond‐Blackfan Anemia Syndrome
Source: Am J Hematol. 2025 Nov 17;101(2):228–41. doi: 10.1002/ajh.70141 (PMC12766366; doi:10.1002/ajh.70141)
Supplement: Supplementary file 1 — Appendix S1: Supporting Information. [file AJH-101-228-s001.docx]

***Supplemental Material***

| **Patient ID** | **DBA#** | **Gender** | **Age at diagnosis *(months)*** | **Age at analysis *(months)*** | **Malformations** | **Malformations (details)** | **Initial response to steroids** | **Neoplasia** | **Follow up** | **Age at last follow up *(years)*** | **Status at last follow up** |
| --- | --- | --- | --- | --- | --- | --- | --- | --- | --- | --- | --- |
| 1 | DBA1 | F | 0 | 4 | Yes | Severe pulmonary valve stenosis | NR |  | Alive | 2.4 | Treatment independence after HSCT |
| 2 | DBA1 | M | 0 | 10 | Yes | Strabismus | CR |  | Alive | 4.8 | Steroid dependence |
| 3 | DBA1 | M | 2 | 72 | Yes | Inguinal hernia | CR |  | Alive | 22.8 | Steroid dependence |
| 4 | DBA1 | F | 1 | 299 | Yes | Craniofacial malformations | CR |  | Alive | 29.3 | Treatment independence after steroids |
| 5 | DBA1 | M | 1 | 2 | Yes | Hydronephrosis, atrial septal defect | NR |  | Alive | 1.5 | Transfusion dependence |
| 6 | DBA1 | M | 1 | 95 | None |  | NR |  | Alive | 19.7 | Treatment independence after HSCT |
| 7 | DBA1 | F | 3 | 4 | None |  | CR |  | Alive | 12.4 | Steroid dependence |
| 8 | DBA1 | M | 0 | 49 | Yes | Auricular fistula, congenital glaucoma, flat thenar eminence, inguinal hernia | CR | Bone sarcoma | Alive | 23.7 | Treatment independence after HSCT |
| 9 | DBA1 | M | 5 | 163 | None |  | NR |  | Alive | 18.1 | Spontaneous treatment independence |
| 10 | DBA1 | M | 2 | 4 | None |  | PR |  | Alive | 8.4 | Treatment independence after HSCT |
| 11 | DBA1 | F | 1 | 4 | None |  | NP |  | Death | 19.1 | // |
| 12 | DBA1 | F | 8 | 8 | Yes | Bifid uvula | NR | Bone sarcoma | Alive | 17.9 | Treatment independence after HSCT |
| 13 | DBA1 | F | 0 | 98 | Yes | Craniofacial dysmorphism, congenital deafness | NR |  | Alive | 14.9 | Treatment independence after HSCT |
| 14 | DBA1 | F | 1 | 329 | Yes | Thumb duplication | CR |  | Alive | 37.0 | Treatment independence after steroids |
| 15 | DBA1 | M | 3 | 24 | None |  | CR |  | Alive | 17.6 | Treatment independence after steroids |
| 16 | DBA1 | M | 28 | 29 | None |  | NR |  | Alive | 14.4 | Treatment independence after HSCT |
| 17 | DBA1 | M | 18 | 175 | Yes | Learning difficulties | CR |  | Alive | 14.3 | Steroid dependence |
| 18 | DBA1 | M | 0 | 0 | Yes | Duplicated collecting system | CR |  | Alive | 3.3 | Steroid dependence |
| 19 | DBA1 | M | 13 | 19 | None |  | NP |  | Alive | 5.9 | Lost at last follow up |
| 20 | DBA1 | F | 1 | 2 | Yes | Facial dysmorphisms, supernumerary nipple, learning difficulties | NR |  | Alive | 19.3 | Treatment independence after HSCT |
| 21 | DBA1 | M | 0 | 2 | Yes | Hydrops fetalis | NR |  | Alive | 6.9 | Transfusion dependence |
| 22 | DBA1 | F | 0 | 4 | None |  | NR |  | Alive | 1.1 | Transfusion dependence |
| 23 | DBA1 | M | 1 | 254 | Yes |  | NR |  | Alive | 18.1 | Transfusion dependence |
| 24 | DBA1 | F | 29 | 394 | None |  | NR |  | Alive | 52.1 | Transfusion dependence |
| 25 | DBA1 | M | 2 | 356 | Yes | Flat thenar eminence | NR |  | Alive | 41.7 | Spontaneous treatment independence |
| 26 | DBA1 | M | 0 | 24 | None |  | NP |  | Alive | 0.0 | Lost at last follow up |
| 27 | DBA1 | M | 2 | 5 | Yes | Patent foramen ovale | CR |  | Alive | 2.6 | Steroid dependence |
| 28 | DBA1 | M | 3 | 5 | Yes | Hypertelorism, facial dysmorphism, left pyelectasis, developmental delay | CR |  | Alive | 7.3 | Steroid dependence |
| 29 | DBA1 | F | 0 | 215 | None |  | CR |  | Alive | 24.2 | Steroid dependence |
| 30 | DBA1 | F | 1 | 8 | None |  | NP | Bone sarcoma | Death | 9.8 | // |
| 31 | DBA4 | M | 4 | 61 | Yes | Cryptorchidism, inguinal hernia | NR |  | Alive | 4.7 | Transfusion dependence |
| 32 | DBA4 | F | 13 | 367 | None |  | CR |  | Alive | 29.9 | Treatment independence after steroids |
| 33 | DBA4 | M | 4 | 350 | None |  | CR |  | Alive | 29.8 | Treatment independence after steroids |
| 34 | DBA4 | M | 3 | 125 | None |  | NP |  | Alive | 3.2 | Spontaneous treatment independence |
| 35 | DBA4 | M | 2 | 329 | None |  | CR |  | Alive | 35.8 | Treatment independence after steroids |
| 36 | DBA4 | M | 3 | 373 | None |  | NR | Thyroid adenoma | Alive | 36.2 | Transfusion dependence |
| 37 | DBA5 | M | 54 | 54 | None |  | NR |  | Alive | 9.2 | Treatment independence after HSCT |
| 38 | DBA5 | F | 0 | 4 | None |  | NR |  | Alive | 13.9 | Treatment independence after HSCT |
| 39 | DBA5 | M | 0 | 34 | None |  | PR |  | Alive | 2.7 | Transfusion dependence |
| 40 | DBA5 | M | 0 | 6 | Yes | Low set ears, patent foramen ovale, imperforated anus, mild developmental delay | NR |  | Alive | 3.2 | Treatment independence after HSCT |
| 41 | DBA5 | F | 20 | 36 | None |  | NR |  | Alive | 6.0 | Treatment independence after HSCT |
| 42 | DBA5 | F | 5 | 226 | None |  | CR |  | Alive | 26.5 | Transfusion dependence |
| 43 | DBA5 | M | 12 | 12 | None |  | NP | MDS | Alive | 10.6 | Treatment independence after HSCT |
| 44 | DBA5 | M | 4 | 25 | Yes | Intrauterine growth retardation, broad flat nasal bridge | NP |  | Alive | 8.2 | Spontaneous treatment independence |
| 45 | DBA6 | M | 0 | 4 | None |  | CR |  | Alive | 4.0 | Steroid dependence |
| 46 | DBA6 | F | 2 | 82 | None |  | CR |  | Alive | 10.4 | Steroid dependence |
| 47 | DBA6 | F | 3 | 155 | Yes | Cleft palate, triphalangeal thumb | CR |  | Alive | 12.7 | Steroid dependence |
| 48 | DBA6 | F | 3 | 16 | Yes | Flat thenar eminence | PR |  | Alive | 12.3 | Transfusion dependence |
| 49 | DBA6 | F | 1 | 4 | Yes | Cleft palate | NP |  | Alive | 3.2 | Lost at last follow up |
| 50 | DBA6 | F | 209 | 209 | None |  | NP |  | Alive | 18.7 | Spontaneous treatment independence |
| 51 | DBA6 | F | 1 | 354 | Yes | Cleft palate | CR |  | Alive | 51.0 | Steroid dependence |
| 52 | DBA6 | M | 0 | 12 | Yes | Craniofacial malformations, low set ears, bifid uvula, cryptorchidism, café au lait spot | PR |  | Alive | 7.3 | Treatment independence after HSCT |
| 53 | DBA6 | M | 2 | 56 | Yes | Facial dysmorphisms, strabismus | CR |  | Alive | 9.1 | Steroid dependence |
| 54 | DBA6 | F | 0 | 2 | Yes | Micrognathia, microcephaly, cleft palate, broad flat nasal bridge, hypertelorism, bifid uvula, mild developmental delay | NP |  | Alive | 5.0 | Transfusion dependence |
| 55 | DBA6 | F | 4 | 90 | Yes | Hypertelorism, broad flat nasal bridge, triphalangeal thumb, flat thenar eminence | NP |  | Alive | 7.4 | Spontaneous treatment independence |
| 56 | DBA6 | M | 5 | 244 | None |  | NR |  | Alive | 24.5 | Transfusion dependence |
| 57 | DBA6 | M | 3 | 160 | Yes | Cleft palate, triphalangeal thumb, agenesis of the corpus callosum | PR |  | Alive | 16.4 | Transfusion dependence |
| 58 | DBA6 | M | 4 | 257 | Yes | Inguinal hernia | PR |  | Alive | 26.9 | Transfusion dependence |
| 59 | DBA6 | F | 0 | 398 | Yes | Craniofacial malformations, flat thenar eminence | CR | NHL | Alive | 53.6 | Steroid dependence |
| 60 | DBA7 | F | 0 | 377 | Yes | Cathie's face, flat thenar eminence | CR |  | Alive | 45.3 | Transfusion dependence |
| 61 | DBA7 | M | 1 | 102 | Yes | Cleft palate, micrognathia, triphalangeal thumb | CR |  | Death  (after HSCT) | 15.7 | // |
| 62 | DBA7 | M | 2 | 222 | Yes | Tetralogy of Fallot, flat thenar eminence hypoplasia | PR |  | Alive | 25.7 | Transfusion dependence |
| 63 | DBA7 | F | 50 | 72 | Yes | Ventricular septal defect, hypoplasia of the first metacarpal ray | NR |  | Alive | 16.9 | Treatment independence after HSCT |
| 64 | DBA7 | M | 2 | 20 | Yes | Hydrops fetalis, ambiguous genitalia | NR |  | Alive | 9.8 | Transfusion dependence |
| 65 | DBA7 | M | 3 | 179 | None |  | NR |  | Alive | 19.9 | Transfusion dependence |
| 66 | DBA7 | F | 2 | 238 | Yes | Tetralogy of Fallot, flat thenar eminence | NP |  | Alive | 38.6 | Spontaneous treatment independence |
| 67 | DBA7 | F | 14 | 70 | None |  | CR |  | Alive | 23.2 | Transfusion dependence |
| 68 | DBA7 | M | 7 | 10 | Yes | Flat thenar eminence | CR |  | Alive | 1.4 | Steroid dependence |
| 69 | DBA7 | F | 1 | 1 | Yes | Triphalangeal thumb | CR |  | Alive | 3.8 | Steroid dependence |
| 70 | DBA7 | F | 3 | 6 | Yes | Atrial septal defect, flat thenar eminence | NR |  | Alive | 7.2 | Treatment independence after HSCT |
| 71 | DBA7 | F | 2 | 217 | None |  | NR |  | Death (after HSCT) | 24.8 | // |
| 72 | DBA7 | M | 27 | 38 | Yes | Flat thenar eminence | CR |  | Alive | 3.1 | Steroid dependence |
| 73 | DBA10 | F | 1 | 203 | Yes | Ostium secundum atrial septal defect | NR |  | Alive | 21.9 | Transfusion dependence |
| 74 | DBA10 | M | 2 | 4 | None |  | CR |  | Alive | 2.4 | Steroid dependence |
| 75 | DBA10 | M | 4 | 149 | None |  | NR |  | Alive | 17.4 | Transfusion dependence |
| 76 | DBA10 | F | 1 | 1 | Yes | Ventricular septal defect | NR |  | Death (after HSCT) | 19.9 | // |
| 77 | DBA10 | F | 11 | 16 | None |  | NP |  | Alive | 15.5 | Treatment independence after HSCT |
| 78 | DBA10 | F | 8 | 11 | None |  | CR |  | Alive | 21.5 | Spontaneous treatment independence |
| 79 | DBA10 | M | 3 | 559 | None |  | CR |  | Alive | 47.9 | Steroid dependence |
| 80 | DBA10 | M | 0 | 6 | Yes | Cleft palate, low set ears, strabismus, hydronephrosis | NP |  | Alive | 1.7 | Lost at last follow up |

**Table S1. Clinical features of DBA cases with molecular diagnosis.**

In case of HSCT, methylation analysis was performed on DNA collected before HSCT.

Legend. M: male; F: female; NR: no response; PR: partial response; CR: complete response; NP: not performed; HSCT-human stem cell transplant, MDS: Myelodisplasia, NHL: Non-Hodgkin Lymphoma

| **Patient ID** | **Gender** | **Age at diagnosis (months)** | **Age at analysis (months)** | **Malformations** | **Malformations (details)** | **Initial response to steroids** | **Neoplasia** | **Follow up** | **Age at last follow up**  **(years)** | **Status at last follow up** | **eADA^** | **rRNA 28S/18S°** | **rRNA 32S/28S°°** | **MVP score** |
| --- | --- | --- | --- | --- | --- | --- | --- | --- | --- | --- | --- | --- | --- | --- |
| 81 | F | 0 | 19 | None |  | NP | None | Alive | 15.4 | Treatment independence after HSCT | 9.3 | NP | NP | 1 |
| 82 | M | 0 | 6 | Yes | Facial dysmorphisms, agenesis of the first left metacarpophalangeal ray, hypospadias | CR | None | Alive | 10.0 | Steroid dependence | 5.09 | 1.67 | 0.090 | 0.99 |
| 83 | F | 3 | 242 | Yes | Thumb malformation, right pyelectasis | CR | None | Alive | 18.2 | Steroid dependence | 4 | 1.65 | 0.027 | 1 |
| 84 | M | 2 | 438 | None |  | CR | None | Alive | 47.7 | Spontaneous treatment independence | 5.86 | 1.82 | 0.025 | 0.02 |
| 85 | M | 11 | 241 | None |  | CR | None | Alive | 23.3 | Treatment independence after steroids | 5 | 1.8 | 0.026 | 0.09 |
| 86 | F | 11 | 341 | Yes | Thumb malformation | CR | None | Alive | 27.9 | Treatment independence after steroids | 5.3 | 1.78 | 0.022 | 0.02 |

**Table S2. Clinical characteristics of DBA patients without molecular diagnosis**

In case of HSCT, methylation analysis was performed on DNA collected before HSCT.

Legend. NP: not performed. ^ normal range: ≤1.2 IU/g Hemoglobin. ° rRNA 28S/18S ratio normal value: 1.7-1.9. °° rRNA 32S/28S ratio normal value: <0.05.

| Subtype | Gene | Gene deletion | Intragenic deletion | Intragenic duplication | Frameshift | Missense | Nonsense | Splicing | All |
| --- | --- | --- | --- | --- | --- | --- | --- | --- | --- |
| DBA1 | *RPS19* | 6 | 1 | - | 5 | 12 | 3 | 3 | 30 |
| DBA4 | *RPS17* | 5 | - | - | - | - | - | 1 | 6 |
| DBA5 | *RPL35A* | 6 | - | 1 | - | - | - | 1 | 8 |
| DBA6 | *RPL5* | 2 | - | - | 7 | 1 | 3 | 2 | 15 |
| DBA7 | *RPL11* | 2 | 1 | - | 7 | - | 1 | 2 | 13 |
| DBA10 | *RPS26* | - | - | - | 2 | 2 | 1 | 3 | 8 |
| Total |  | 21 | 2 | 1 | 21 | 15 | 8 | 12 | 80 |

**Table S3. DBA subtype, causative gene, and variant type for 80 individuals**

| **Patient ID** | **DBA#** | **eADA^** | **GENE** | **Coding Change/Genomic Change** | **Protein Change** | **Pathogenicity (ACMG )** | **Inheritance** | **comment** |
| --- | --- | --- | --- | --- | --- | --- | --- | --- |
| 1 | DBA1 | normal* | RPS19 | c.167G>T | p.(Arg56Leu) | likely pathogenic (PM1; PM2; PM5; PP2; PP5) | unknown |  |
| 2 | DBA1 | 2.24 | RPS19 | c.34C>T | p.(Gln12Ter) | pathogenic (PVS1;PS2; PS4; PM2) | *de novo* |  |
| 3 | DBA1 | 3.6 | RPS19 | c.58delG | p.(Ala20ProfsTer9) | pathogenic (PVS1;PS2;PM2) | *de novo* |  |
| 4 | DBA1 | 2.32 | RPS19 | c.3G>T | p.? | pathogenic (PVS1;PS1;PS2;PM2) | *de novo* |  |
| 5 | DBA1 | normal* | RPS19 | g.(?_42364026)_(42375346_?)del | p.? | pathogenic (PVS1;PS2;PM2) | *de novo* |  |
| 6 | DBA1 | normal* | RPS19 | c.167G>A | p.(Arg56Gln) | pathogenic (PS4; PM1; PM2; PM5; PP2) | inherited (mother) | clinical status of  the mother not known |
| 7 | DBA1 | 1.98 | RPS19 | c.301dupC | p.(Arg101ProfsTer53) | pathogenic (PVS1;PS2;PM2) | *de novo* |  |
| 8 | DBA1 | 5.4 | RPS19 | c.184C>T | p.(Arg62Trp) | pathogenic (PS3;PS2; PM1; PM2; PM5; PP2) | *de novo* |  |
| 9 | DBA1 | 3.12 | RPS19 | c.357-1G>T | p.? | likely pathogenic (PVS1;PM2) | unknown |  |
| 10 | DBA1 | NP | RPS19 | c.411+1G>A | p.? | pathogenic (PVS1;PS2;PM2) | *de novo* |  |
| 11 | DBA1 | NP | RPS19 | g.(?_42364026)_(42375346_?)del | p.? | pathogenic (PVS1;PS2;PM2) | *de novo* |  |
| 12 | DBA1 | normal* | RPS19 | c.1-1G>A | p.? | pathogenic (PVS1;PS2;PM2) | *de novo* |  |
| 13 | DBA1 | normal* | RPS19 | c.184C>T | p.(Arg62Trp) | pathogenic (PM1;PS2;PM2;PM5; PP2) | *de novo* |  |
| 14 | DBA1 | 2.65 | RPS19 | c.156G>C | p.(Trp52Cys) | likely pathogenic (PM1;PM2;PP2;PP3) | unknown |  |
| 15 | DBA1 | 3.15 | RPS19 | c.383_384delAA | p.(Asp130SerfsTer23) | pathogenic (PVS1;PS2;PS3;PS4;PM2) | *de novo* |  |
| 16 | DBA1 | normal* | RPS19 | c.53T>C | p.(Leu18Pro) | likely pathogenic (PS3; PM1; PM2; PM5; PP2;PP3) | unknown |  |
| 17 | DBA1 | 1.95 | RPS19 | c.-235_144del (ex1-3) | p.? | likely pathogenic (PVS1;PM2) | unknown |  |
| 18 | DBA1 | 1.73 | RPS19 | c.185G>A | p.(Arg62Gln) | pathogenic (PS3; PS4; PM1; PM2; PM5; PP2) | inherited (father) | affected |
| 19 | DBA1 | 2.88 | RPS19 | c.301delC | p.(Arg101AlafsTer10) | pathogenic (PVS1;PS2;PM2) | *de novo* |  |
| 20 | DBA1 | 3.8 | RPS19 | c.412delG | p.(Val138Trpfs) | pathogenic (PVS1;PS2;PM2) | *de novo* |  |
| 21 | DBA1 | normal* | RPS19 | g.(?_42364026)_(42375346_?)del | p.? | likely pathogenic | *de novo* |  |
| 22 | DBA1 | 1.9* | RPS19 | g.(?_42364026)_(42375346_?)del | p.? | likely pathogenic | *de novo* |  |
| 23 | DBA1 | normal* | RPS19 | g.(?_42364026)_(42375346_?)del | p.? | likely pathogenic | *de novo* |  |
| 24 | DBA1 | NP | RPS19 | c.280C>T | p.(Arg94Ter) | pathogenic (PVS1; PS3;PS4;PM2) | unknown |  |
| 25 | DBA1 | 3/0.45** | RPS19 | c.140C>T | p.(Pro47Leu) | pathogenic (PS2; PS3; PS4; PM2; PP2;PP3) | *de novo* | revertant case |
| 26 | DBA1 | NP | RPS19 | c.140C>T | p.(Pro47Leu) | likely pathogenic (PS3;PS4; PM2; PP2: PP3) | unknown |  |
| 27 | DBA1 | normal* | RPS19 | c.65T>C | p.(Leu22Pro) | likely pathogenic (PS2; PM1; PM2; PP2; PP3) | *de novo* |  |
| 28 | DBA1 | 2.42 | RPS19 | g.(?_42364026)_(42375346_?)del | p.? | likely pathogenic | *de novo* |  |
| 29 | DBA1 | 2.4 | RPS19 | c.403G>A | p.(Ala135Thr) | likely pathogenic (PS2; PM2; PP2; PP5) | *de novo* |  |
| 30 | DBA1 | 4.7 | RPS19 | c.166C>T | p.(Arg56Ter) | pathogenic (PVS1; PS4; PM2) | unknown |  |
| 31 | DBA4 | normal* | RPS17 | g.(?_83205447)_(83209177_?)del | p.? | likely pathogenic | *de novo* |  |
| 32 | DBA4 | 1.43 | RPS17 | g.(?_83205447)_(83209177_?)del | p.? | likely pathogenic | unknown |  |
| 33 | DBA4 | 1.7 | RPS17 | g.(?_83205447)_(83209177_?)del | p.? | likely pathogenic | unknown |  |
| 34 | DBA4 | 3.47 | RPS17 | c.155+1G>A | p.? | pathogenic (PVS1;PS2;PM2) | *de novo* |  |
| 35 | DBA4 | 0.86** | RPS17 | g.(?_83205447)_(83209177_?)del | p.? | likely pathogenic | unknown | revertant case |
| 36 | DBA4 | NP | RPS17 | g.(?_83205447)_(83209177_?)del | p.? | likely pathogenic | inherited (mother) | clinical status of  the mother not known |
| 37 | DBA5 | 2.35 | RPL35A | g.(?_197677041)_(197682761_?)del | p.? | likely pathogenic | unknown |  |
| 38 | DBA5 | NP | RPL35A | g.(?_197677041)_(197682761_?)del | p.? | likely pathogenic | *de novo* |  |
| 39 | DBA5 | normal* | RPL35A | g.(?_197677041)_(197682761_?)del | p.? | likely pathogenic | unknown |  |
| 40 | DBA5 | normal* | RPL35A | g.(?_197677041)_(197682761_?)del | p.? | likely pathogenic | *de novo* |  |
| 41 | DBA5 | normal* | RPL35A | c.165-759_310-62dup | p.(Met104GlnfsTer40) | likely pathogenic | unknown |  |
| 42 | DBA5 | 1.55 | RPL35A | g.(?_197677041)_(197682761_?)del | p.? | likely pathogenic | *de novo* |  |
| 43 | DBA5 | 2.65 | RPL35A | g.(?_197677041)_(197682761_?)del | p.? | likely pathogenic | unknown |  |
| 44 | DBA5 | 4.1 | RPL35A | c.164+5G>A | p.? | likely pathogenic (PS2; PM2; PP3) | *de novo* |  |
| 45 | DBA6 | 5.28 | RPL5 | c.175_176delGA | p.(Asp59TyrfsTer53) | pathogenic(PVS1; PS2; PS4; PM2) | *de novo* |  |
| 46 | DBA6 | 3.67 | RPL5 | c.3G>C | p.Met1? | pathogenic (PVS1; PS1; PS2; PM2) | *de novo* |  |
| 47 | DBA6 | 2.75 | RPL5 | c.535C>T | p.(Arg179Ter) | likely pathogenic (PVS1; PM2) | inherited (mother) | affected |
| 48 | DBA6 | 7.9 | RPL5 | c.678C>A | p.(Tyr226Ter) | pathogenic (PVS1; PS2; PM2) | *de novo* |  |
| 49 | DBA6 | 6.86 | RPL5 | c.42delG | p.(Arg15AspfsTer4) | likely pathogenic (PVS1; PM2) | unknown |  |
| 50 | DBA6 | 2.32 | RPL5 | c.3+5G>A | p.? | likely pathogenic (PS2; PM2; PP3) | *de novo* |  |
| 51 | DBA6 | 2.58 | RPL5 | c.134_138delACACA | p.(Asn45ThrfsTer66) | pathogenic (PVS1; PS2; PM2) | *de novo* |  |
| 52 | DBA6 | normal* | RPL5 | g.(?_93297669)_(93307645_?)del | p.? | likely pathogenic | *de novo* |  |
| 53 | DBA6 | 2.95 | RPL5 | c.39_40insT | p.(Lys14Ter) | pathogenic (PVS1; PS2; PM2) | *de novo* |  |
| 54 | DBA6 | 4.5 | RPL5 | c.633_636delAATG | p.(Met212LysfsTer15) | pathogenic (PVS1; PS2; PM2) | *de novo* |  |
| 55 | DBA6 | 4.7 | RPL5 | c.91delT | p.(Tyr31MetfsTer7) | pathogenic (PVS1; PS2; PM2) | *de novo* |  |
| 56 | DBA6 | NP | RPL5 | c.189+1G>A | p.? | likely pathogenic (PVS1; PM2) | unknown |  |
| 57 | DBA6 | normal* | RPL5 | c.172_173insA | p.(Arg58LysfsTer55) | pathogenic (PVS1; PS2; PM2) | *de novo* |  |
| 58 | DBA6 | 2,32* | RPL5 | c.283delT | p.(Tyr95MetfsTer31) | pathogenic (PVS1; PS2; PM2) | *de novo* |  |
| 59 | DBA6 | 6.6 | RPL5 | g.(?_93297669)_(93307645_?)del | p.? | likely pathogenic | *de novo* |  |
| 60 | DBA7 | 3.79 | RPL11 | c.198_202delAAAGA | p.(Glu66AspfsTer26) | likely pathogenic (PVS1; PM2) | unknown |  |
| 61 | DBA7 | 2.71 | RPL11 | c.469delA | p.(Ile157SerfsTer37) | pathogenic (PVS1; PS2; PM2) | *de novo* |  |
| 62 | DBA7 | 3,2* | RPL11 | c.314_315delTT | p.(Phe105TrpfsTer15) | pathogenic (PVS1; PS2; PM2) | *de novo* |  |
| 63 | DBA7 | 4.58 | RPL11 | c.143_157+32del | p.? | likely pathogenic (PVS1; PM2) | unknown |  |
| 64 | DBA7 | 1,48* | RPL11 | g.(?_24018319)_(24022925_?)del | p.? | likely pathogenic | inherited (father) | affected |
| 65 | DBA7 | 1.76* | RPL11 | c.95_96delGA | p.(Arg32ThrfsTer22) | pathogenic(PVS1; PS4; PM2) | unknown |  |
| 66 | DBA7 | 6.06 | RPL11 | g.(?_24022395)_(24022925_?)del (ex5-6) | p.? | likely pathogenic | *de novo* |  |
| 67 | DBA7 | NP | RPL11 | c.60_61delCT | p.(Cys21SerfsTer33) | pathogenic(PVS1; PS2; PM2) | inherited (mother) | clinical status of  the mother not known |
| 68 | DBA7 | 3.74 | RPL11 | c.433A>T | p.(Lys145Ter) | pathogenic (PVS1; PS2; PM2) | *de novo* |  |
| 69 | DBA7 | NP | RPL11 | g.(?_24018319)_(24022925_?)del | p.? | likely pathogenic | unknown |  |
| 70 | DBA7 | 2,02* | RPL11 | c.465_466delCA | p.(His155GlnfsTer16) | pathogenic(PVS1; PS2; PM2) | *de novo* |  |
| 71 | DBA7 | 4,4* | RPL11 | c.508-2A>G | p.? | likely pathogenic (PVS1; PM2) | inherited (father) | affected |
| 72 | DBA7 | 2.84 | RPL11 | c.444_445dupCA | p.(Gly149GlnfsTer46) | likely pathogenic (PVS1; PM2) | inherited (mother) | affected |
| 73 | DBA10 | normal* | RPS26 | c.3+2T>A | p.? | pathogenic (PVS1; PS2; PM2) | *de novo* |  |
| 74 | DBA10 | 2* | RPS26 | c.1A>G | p.? | pathogenic (PVS1; PS1; PS2; PM2) | *de novo* |  |
| 75 | DBA10 | normal* | RPS26 | c.78_79delTG | p.(Ala27ProfsTer10) | pathogenic (PVS1; PM2; PP5) | unknown |  |
| 76 | DBA10 | NP | RPS26 | c.4-1G>A | p.? | likely pathogenic (PVS1; PM2) | inherited (mother) | clinical status of  the mother not known |
| 77 | DBA10 | NP | RPS26 | c.222_225delTGTG | p.(Cys74Ter) | pathogenic (PVS1; PS2; PM2) | de novo |  |
| 78 | DBA10 | 3.12 | RPS26 | c.3G>A | p.? | pathogenic (PVS1; PS2; PM2) | de novo |  |
| 79 | DBA10 | 1.18 | RPS26 | c.50dupA | p.(His17GlnfsTer9) | likely pathogenic (PVS1; PM2) | unknown |  |
| 80 | DBA10 | 3.55 | RPS26 | c.307_312+4del | p.? | likely pathogenic (PVS1; PM2) | unknown |  |

**Table S4. ACMG variant classifications in DBA patients**

Legend. Genomic reference sequence for all genes: Human GRCh37/hg19. Coding reference sequence: NM_001022.4 for *RPS19*; NM_001021.6 for *RPS17*; NM_000996.4 for *RPL35A*; NM_000969.5 for *RPL5*; NM_000975.5 for *RPL11*; NM_001029.5 for *RPS26.* ^ normal range: ≤1.2 IU/g Hemoglobin. *: eADA values in patients receiving transfusions. ** eADA values in patients with molecular reversion.

| **Signature name** | **Gene or region** | **Disorder abbreviation(s)** | **Disorder** | **OMIM number(s)** |
| --- | --- | --- | --- | --- |
| ARID1A_dup | *ARID1A* | [ARID1A_dup] | ARID1A duplication-related syndrome | --- |
| ARTHS | *KAT6A* | ARTHS | Arboleda-Tham syndrome | 616268 |
| ATRX | *ATRX* | ATRX | Alpha-thalassemia/impaired intellectual development syndrome | 301040 |
| BAFopathy | *ARID1A; ARID1B; SMARCA2; SMARCA4; SMARCB1* | CSS1; CSS2; CSS3; CSS4; NCBRS | Coffin-Siris syndrome 1; Coffin-Siris syndrome 2; Coffin-Siris syndrome 3; Coffin-Siris syndrome 4; Nicolaides-Baraitser syndrome | 135900, 614607, 614608, 614609, 601358 |
| BEFAHRS | *TET3* | BEFAHRS | Beck-Fahrner syndrome | 618798 |
| BFLS | *PHF6* | BFLS | Börjeson-Forssman-Lehmann syndrome | 301900 |
| BIS | *SMARCA2* | BIS | Blepharophimosis-impaired intellectual disability syndrome | 619293 |
| CdLS | *NIPBL; RAD21; SMC3; SMC1A* | CDLS1; CDLS2; CDLS3; CDLS4 | Cornelia de Lange syndrome 1; Cornelia de Lange syndrome 2; Cornelia de Lange syndrome 3; Cornelia de Lange syndrome 4 | 122470, 300590, 610759, 614701 |
| CdLS1 | *NIPBL* | CDLS1 | Cornelia de Lange syndrome 1 | 122470 |
| CdLS2 | *SMC1A* | CDLS2 | Cornelia de Lange syndrome 2 | 300590 |
| CdLS3 | *SMC3* | CDLS3 | Cornelia de Lange syndrome 3 | 610759 |
| CdLS4 | *RAD21* | CDLS4 | Cornelia de Lange syndrome 4 | 614701 |
| CHARGE | *CHD7* | HHS | CHARGE syndrome | 214800 |
| CHDFIDD | *CDK13; CCNK* | CHDFIDD | Congenital heart defects, dysmorphic facial features, and intellectual developmental disorder; CCNK-related disorder | 617360 |
| Chr1p36_del | *Chr1p36 deletion* | Chr1p36_del | Chromosome 1p36 deletion syndrome |  |
| ChrXp11.22_dup | *ChrXp11.22 duplication* | [ChrXp11.22_dup] | Chromosome Xp11.22 duplication syndrome | --- |
| CHU_BFL_WHI | *PHIP+PHF6+DDB1* | CHUJANS; BFLS; WHIKERS | Chung-Jansen syndrome; Börjeson-Forssman-Lehmann syndrome; White-Kernohan syndrome | 617991; 301900; 619426 |
| CHUJANS | *PHIP* | CHUJANS | Chung-Jansen syndrome | 617991 |
| CLABARS | *TRIP12* | CLABARS | Clark-Baraitser syndrome | 617752 |
| CSS_c.6200 | *ARID1A; ARID1B* | CSS1; CSS2 | Coffin-Siris syndrome 1; Coffin-Siris syndrome 2 | 135900, 614607 |
| CSS1 | *ARID1B* | CSS1 | Coffin-Siris syndrome 1 | 135900 |
| CSS2 | *ARID1A* | CSS2 | Coffin-Siris syndrome 2 | 614607 |
| CSS3 | *SMARCB1* | CSS3 | Coffin-Siris syndrome 3 | 614608 |
| CSS4 | *SMARCA4* | CSS4 | Coffin-Siris syndrome 4 | 614609 |
| CSS4_c.2656 | *SMARCA4* | CSS4 | Coffin-Siris syndrome 4 | 614609 |
| CSS6 | *ARID2* | CSS6 | Coffin-Siris syndrome 6 | 617808 |
| DEE54 | *HNRNPU* | DEE54 | Developmental and epileptic encephalopathy 54 | 617391 |
| DEE94 | *CHD2* | DEE94 | Developmental and epileptic encephalopathy 94 | 615369 |
| DEGCAGS | *ZNF699* | DEGCAGS | DEGCAGS syndrome | 619488 |
| DIJOS | *KDM3B* | DIJOS | Diets-Jongmans syndrome | 618846 |
| Down | *Chr21 trisomy* | [Down] | Down syndrome | 190685 |
| Dup7 | *Chr7q11.23 duplication* | [Dup7] | Williams-Beuren region duplication syndrome | 609757 |
| DYT28 | *KMT2B* | DYT28 | Dystonia 28, childhood-onset | 617284 |
| FANC=FA | *FANCA+FANCC+FANCD2+FANCG+FANCI+FANCL+FANC_?* | FANCA; FANCC; FANCD; FANCG; FANCI; FANCL | Fanconi anemia, complement group A; Fanconi anemia, complement group C; Fanconi anemia, complement group D2; Fanconi anemia, complement group G; Fanconi anemia, complement group I; Fanconi anemia, complement group L | 607139; 613899; 613984; 602956; 611360; 608111 |
| FLHS | *SRCAP* | FLHS | Floating-Harbor syndrome | 136140 |
| FVS | *Independent_FVS* | FVS | Valproate embryopathy, susceptibility to | 609442 |
| GADEVS | *YY1* | GADEVS | Gabriele-de Vries syndrome | 617557 |
| HAFOUS | *USP7* | HAFOUS | Hao-Fountain syndrome | 616863 |
| HMA | *Chr5q35-qter duplication* | [HMA] | Hunter-McAlpine craniosynostosis syndrome | 601379 |
| HVDAS_C | *ADNP* | HVDAS | Helsmoortel-van der Aa syndrome | 615873 |
| HVDAS_T | *ADNP* | HVDAS | Helsmoortel-van der Aa syndrome | 615873 |
| IDDAM | *CHD8* | AUTS18 | Intellectual developmental disorder with autism and macrocephaly | 615032 |
| IDDMOH | *SOX11* | CSS9 | Coffin-Siris syndrome-9 | 615866 |
| IDDSELD | *SETD1B* | IDDSELD | Intellectual developmental disorder with seizures and language delay | 619000 |
| IDDSFTA | *BCL11B* | IDDSFTA | Intellectual developmental disorder with dysmorphic facies, speech delay, and T-cell abnormalities | 618092 |
| Kabuki | *KMT2D; KDM6A* | KABUK1; KABUK2 | Kabuki syndrome 1; Kabuki syndrome 2 | 147920; 300867 |
| Kabuki1 | *KMT2D* | KABUK1 | Kabuki syndrome 1 | 147920 |
| Kabuki2 | *KDM6A* | KABUK2 | Kabuki syndrome 2 | 300867 |
| KBGS | *ANKRD11* | KBGS | KBG syndrome | 148050 |
| KDM2B | *KDM2B* | [KDM2B] | KDM2B-related syndrome | --- |
| KDVS | *KANSL1* | KDVS | Koolen-De Vries syndrome | 610443 |
| Kleefstra | *EHMT1* | KLEFS1 | Kleefstra syndrome 1 | 610253 |
| BCAHH | *KMT2D* | BCAHH | Branchial arch abnormalities, choanal atresia, athelia, hearing loss, and hypothyroidism syndrome | 620186 |
| LLS | *SETD2* | LLS | Luscan-Lumish syndrome | 616831 |
| Chr19p13.13_del | *Chr19p13.13 deletion* | [Chr19p13.13_del] | Chromosome 19p13.13 deletion syndrome | 613638 |
| MKHK_ID4 | *CREBBP; EP300* | MKHK1; MKHK2 | Menke-Hennekam syndrome 1; Menke-Hennekam syndrome 2 | 618332; 618333 |
| MOWS | *ZEB2* | MOWS | Mowat-Wilson syndrome | 235730 |
| MRD21 | *CTCF* | MRD21 | Intellectual developmental disorder, autosomal dominant 21 | 615502 |
| MRD23 | *SETD5* | MRD23 | Intellectual developmental disorder, autosomal dominant 23 | 615761 |
| MRD51 | *KMT5B* | MRD51 | Intellectual developmental disorder, autosomal dominant 51 | 617788 |
| MRD7 | *DYRK1A* | MRD7 | Intellectual developmental disorder, autosomal dominant 7 | 614104 |
| MRXSA | *FAM50A* | MRXSA | Intellectual developmental disorder, X-linked, syndromic, Armfield type | 300261 |
| MRXSCJ | *KDM5C* | MRXSCJ | Intellectual developmental disorder, X-linked, syndromic, Claes-Jensen type | 300534 |
| MRXSCJ_females | *KDM5C* | MRXSCJ | Intellectual developmental disorder, X-linked, syndromic, Claes-Jensen type | 300534 |
| MRXSN | *UBE2A* | MRXSN | Intellectual developmental disorder, X-linked, syndromic, Nascimento-type | 300860 |
| MRXSSR | *SMS* | MRXSSR | Intellectual developmental disorder, X-linked, syndromic, Snyder-Robinson type | 309583 |
| MSL2 | *MSL2* | [MSL2] | MSL2-related syndrome | --- |
| NCBRS | *SMARCA2* | NCBRS | Nicolaides-Baraitser syndrome | 601358 |
| NEDHSIL | *MEF2C* | NEDHSIL | Neurodevelopmental disorder with hypotonia, stereotypic hand movements, and impaired language | 613443 |
| NSD2_dup | *NSD2* | [NSD2_dup] | NSD2 duplication-related syndrome | --- |
| PHMDS | *Chr22q13.3 deletion* | PHMDS | Phelan-McDermid syndrome | 606232 |
| PRC2 | *EZH2; EED* | WVS; COGIS | Weaver syndrome; Cohen-Gibson syndrome | 277590; 617561 |
| PRR12 | *PRR12* | NOC | Neuroocular syndrome | 619539 |
| PTHS | *TCF4* | PTHS | Pitt-Hopkins syndrome | 610954 |
| PTLS | *Chr17p11.2 duplication* | PTLS | Potocki-Lupski syndrome | 610883 |
| RENS1 | *PQBP1* | RENS1 | Renpenning syndrome | 309500 |
| RMNS | *H1-4* | RMNS | Rahman syndrome | 617537 |
| RSTS | *CREBBP; EP300* | RSTS1; RSTS2 | Rubinstein-Taybi syndrome 1; Rubinstein-Taybi syndrome 2 | 180849; 613684 |
| RSTS1 | *CREBBP* | RSTS1 | Rubinstein-Taybi syndrome 1 | 180849 |
| RSTS2 | *EP300* | RSTS2 | Rubinstein-Taybi syndrome 2 | 613684 |
| SBBYSS | *KAT6B* | SBBYSS | Ohdo syndrome, SBBYSS variant | 603736 |
| SIHIWES | *CHD4* | SIHIWES | Sifrim-Hitz-Weiss syndrome | 617159 |
| SLC32A1 | *SLC32A1* | [SLC32A1] | SLC32A1-related syndrome | --- |
| SMS_del | *Chr17p11.2 deletion* | SMS | Smith-Magenis syndrome | 182290 |
| Sotos | *NSD1* | SOTOS | Sotos syndrome | 117550 |
| NEDFBA | *SRSF1* | NEDFBA | Neurodevelopmental disorder with dysmorphic facies and behavioral abnormalities | 620489 |
| TBRS | *DNMT3A* | TBRS | Tatton-Brown-Rahman syndrome | 615879 |
| Turner | *ChrX deletion (45,X)* | [Turner] | Turner syndrome | --- |
| VCFS | *Chr22q11.2 deletion* | VCFS | Velocardiofacial syndrome | 192430 |
| WDSTS | *KMT2A* | WDSTS | Wiedemann-Steiner syndrome | 605130 |
| WHIKERS | *DDB1* | WHIKERS | White-Kernohan syndrome | 619426 |
| WHS | *Chr4p16.13 deletion* | WHS; RAUST | Wolf-Hirschhorn syndrome; Rauch-Steindl syndrome | 194190; 619695 |
| WHSUS | *POGZ* | WHSUS | White-Sutton syndrome | 616364 |
| Williams | *Chr7q11.23 deletion* | WBS | Williams-Beuren syndrome | 194050 |
| WITKOS | *SIN3A* | WITKOS | Witteveen-Kolk syndrome | 613406 |
| WRWF | *ZC4H2* | WRWF | Wieacker-Wolff syndrome | 314580 |
| XLID93 | *BRWD3* | BRWD3 | Intellecutal developmental disorder, X-linked 93 | 300659 |
| XLID97 | *ZNF711* | XLID97 | Intellectual developmental disorder, X-linked 97 | 300803 |
| XXY | *ChrX duplication* | [XXY] | Klinefelter syndrome | --- |

**Table S5. Abbreviations for the listed epigenetic profiles in the EKD**

**
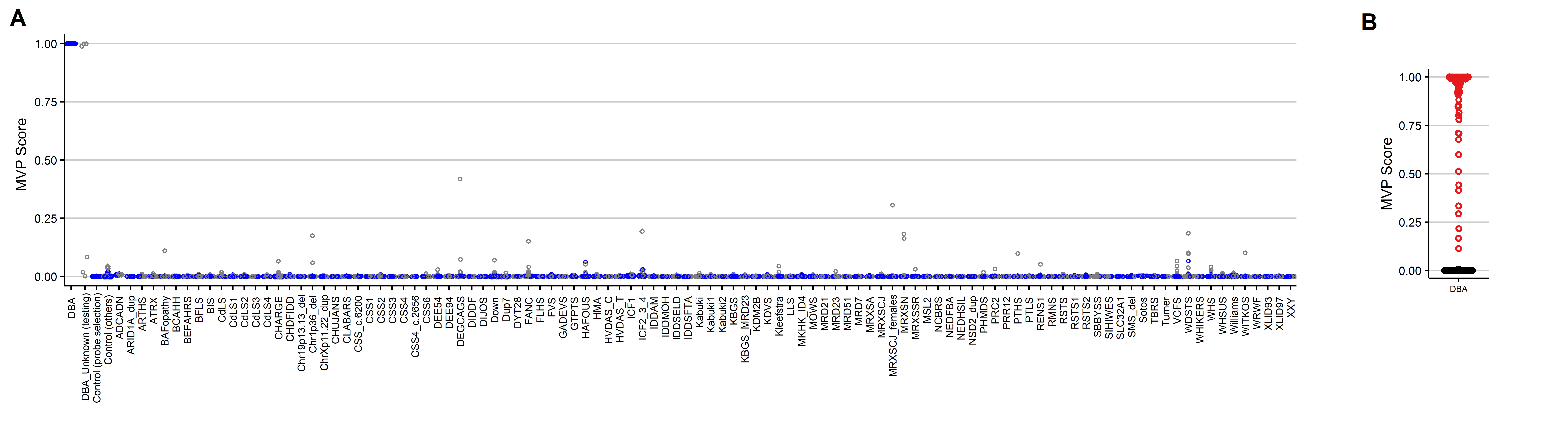
**

**Figure S1.** **Discovery of an episignature in DBAS cohort.**

**(A)** Support Vector Machine classifier: the model was trained by comparing the DBAS cases (n=80) against matched controls, 75% of other controls, and 75% of the other 99 disorders with detectable episignature from the EpiSign Knowledge Database (blue circles). The remaining 25% of controls and 25% of the other 99 disorders with detectable episignature were used for testing (grey circles). The abbreviations for the disorders indicated on X-axis are listed in Supplemental Table 5.

**(B)** Leave-one-out cross-validation: A total of 80 rounds of cross-validation were performed. In each round, one DBAS case was set aside for testing, while the model was trained on the remaining cases. Methylation Variant Pathogenicity (MVP) scores for the testing DBAS cases (red circles), as well as for controls and individuals with other episignature disorders (black circles), were plotted across all rounds. The consistently high MVP scores observed in most DBAS cases confirmed the reproducibility of the episignature.


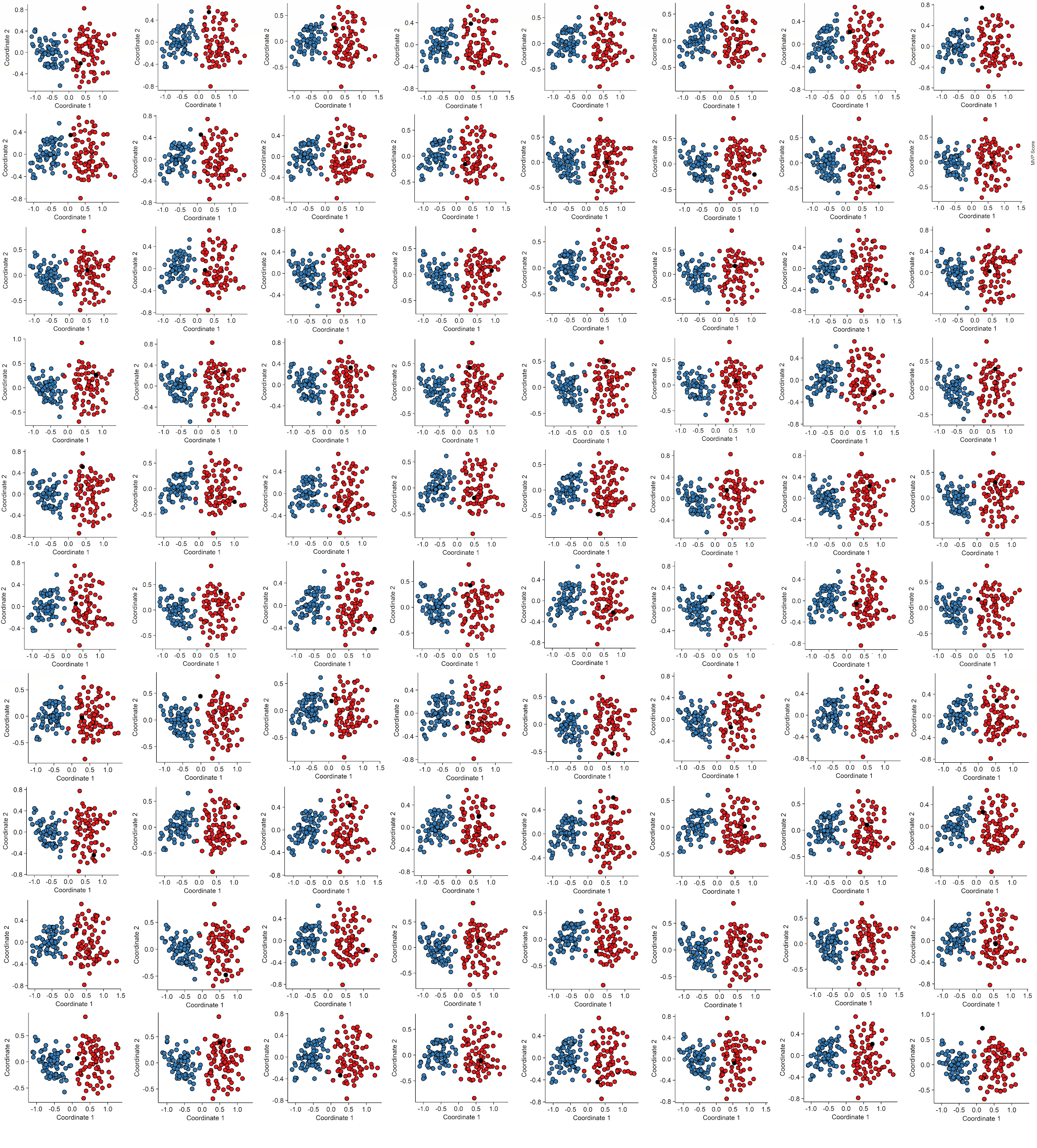


**Figure S2.** **Leave-One-Out Cross-Validation of the DBAS-Episignature.** LOOCV was performed for each sample (n=80) to evaluate the reproducibility and robustness of the DBAS-episignature. In the plots, controls are depicted in blue, DBAS are in red, and the tested case is marked in black. Each plot represents the comparison between the principal components for the respective test case, demonstrating the consistency of the episignature across most samples.


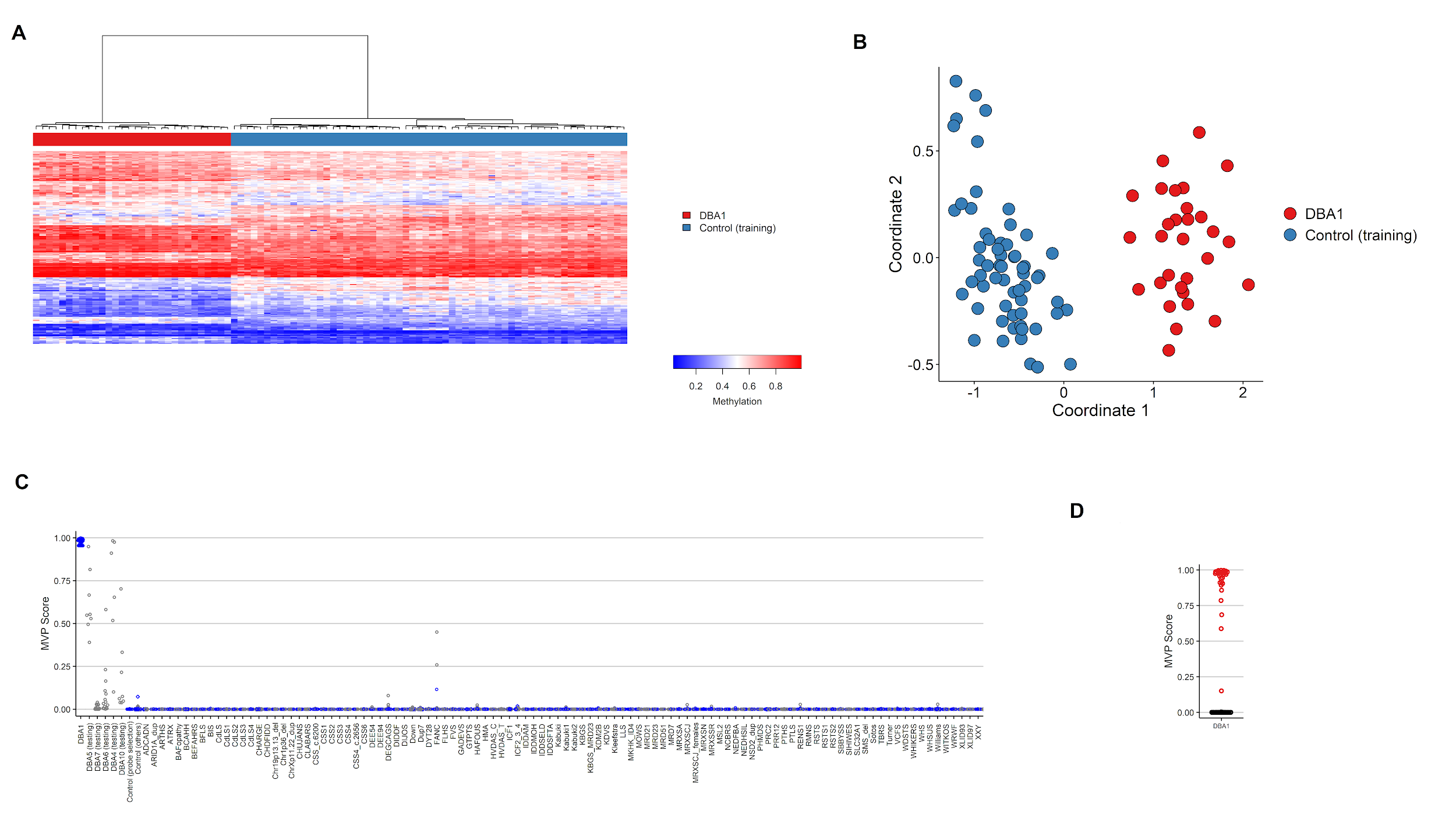


**Figure S3.** **Discovery of an episignature in the DBA1 cohort. (A)** Heatmap illustrating the separation of methylation profiles between DBA1 cases (red) and control samples (blue). **(B)** MDS plot showing the distinct clustering of DBA1 cases (red) and control populations (blue) based on their methylation profiles. **(C)** SVM classifier: the model was trained by comparing DBA1 cases (n=30) to matched controls, with 75% of other controls and 75% of samples from 99 other disorders with detectable episignature (blue circles). Other DBAS cohorts (DBA5, DBA7, DBA6, DBA4, and DBA10) were used as testing sets (gray circles). Additionally, the remaining 25% of controls and 25% of other disorders with detectable episignature were used for testing (gray circles). The abbreviations for the disorders indicated on X-axis are listed in Supplemental Table 5.

**(D)** MVP score distribution for DBA1 cases, showing that most DBA1 cases exhibit high MVP scores.


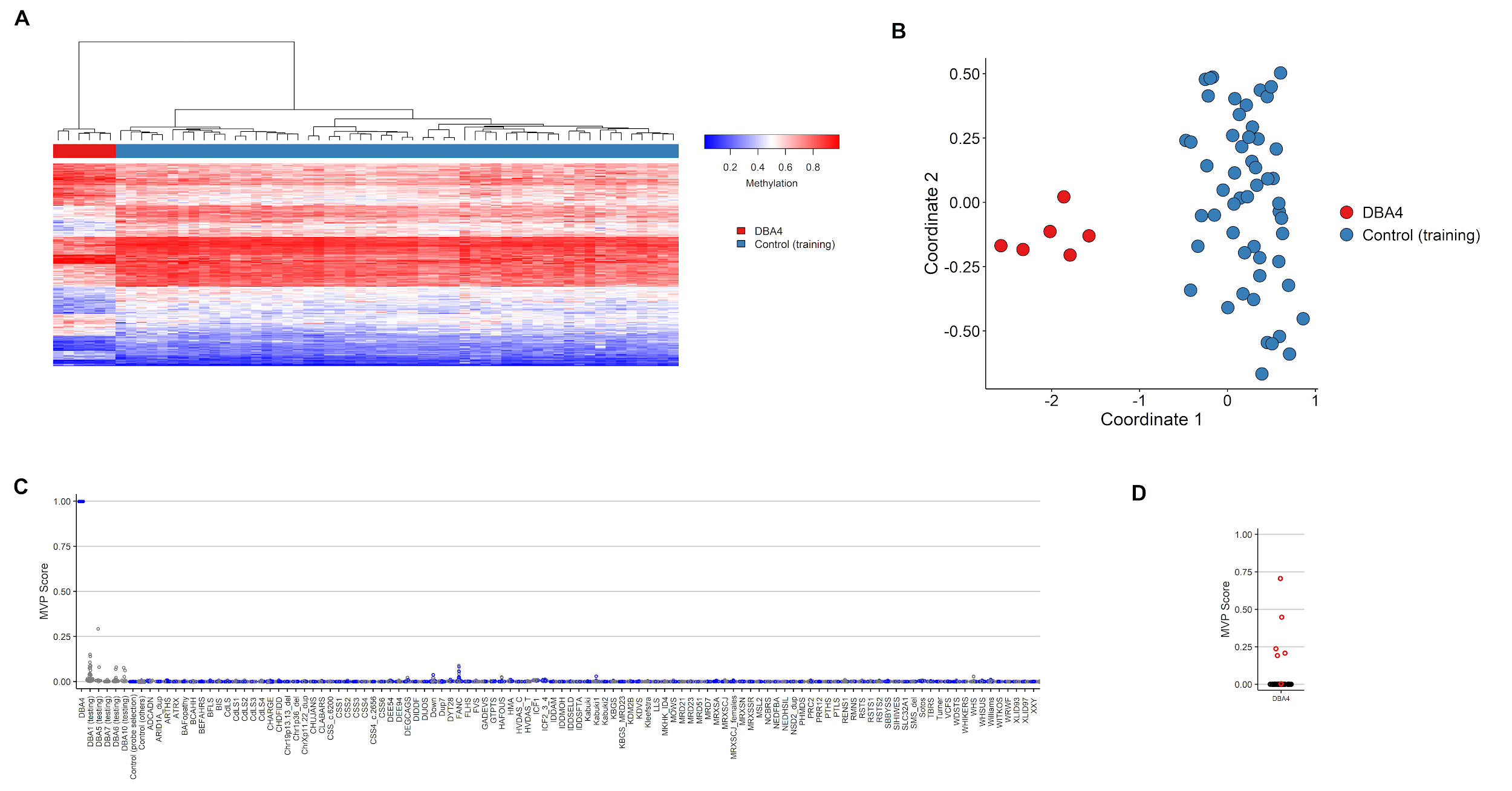


**Figure S4.** **Discovery of an episignature in the DBA4 cohort. (A)** Heatmap showing the separation of methylation profiles between DBA4 cases (red, n=6) and control samples (blue). **(B)** MDS plot illustrating the distinct clustering of DBA4 cases (red) and control populations (blue) based on their methylation profiles. **(C)** MVP scores for DBA4 cases (blue) and control samples (gray), with other DBAS cohorts (DBA1, DBA5, DBA7, DBA6, and DBA10) used as testing sets (gray circles). The abbreviations for the disorders indicated on X-axis are listed in Supplemental Table 5. **(D)** MVP score distribution for DBA4 cases, showing relatively low MVP scores in the DBA4 group.


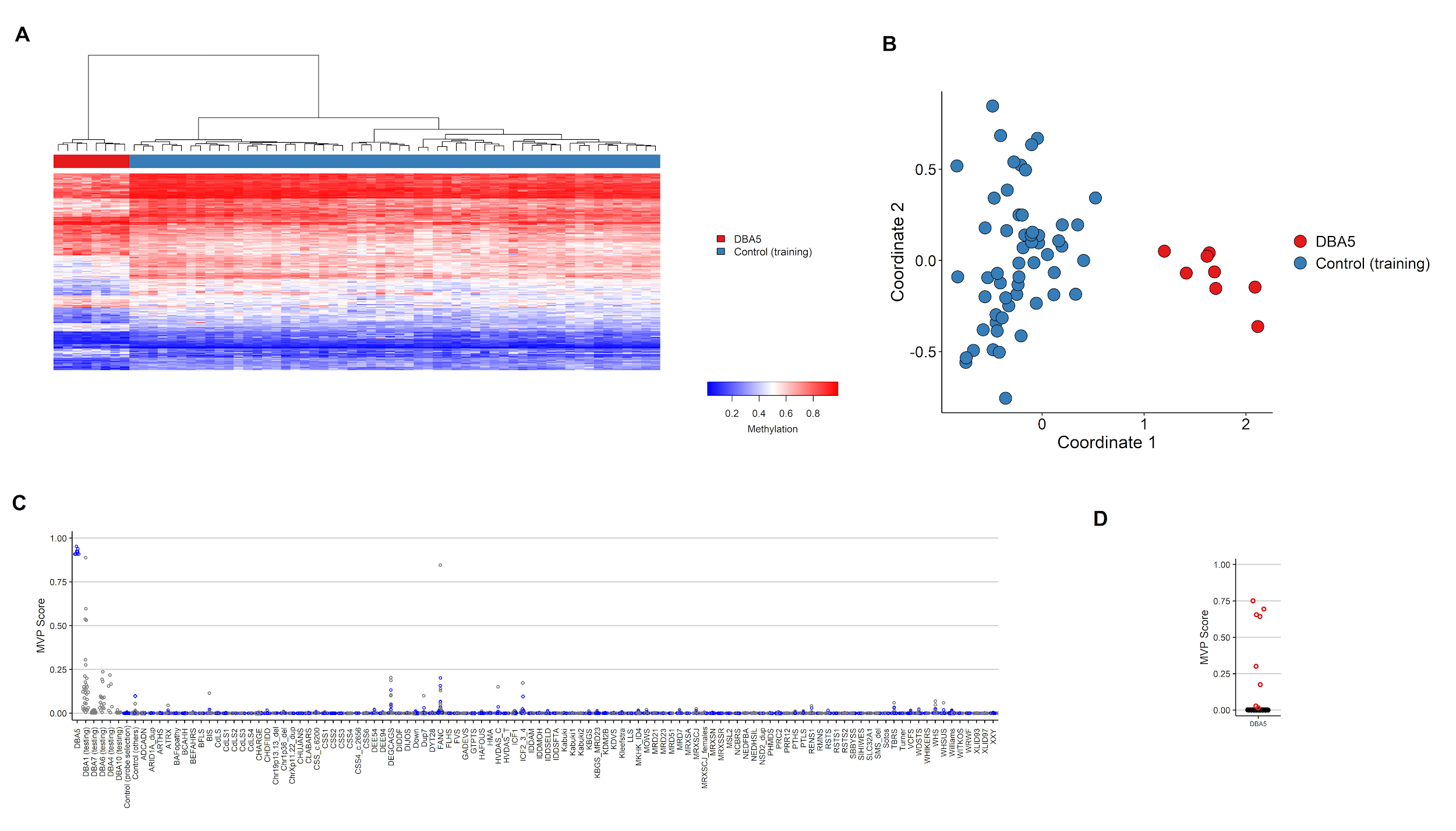


**Figure S5. Identification of an episignature in the DBA5 cohort. (A)** Heatmap displaying the distinct methylation patterns between DBA5 cases (red, n=8) and control samples (blue). **(B)** MDS plot showing the clustering of DBA5 cases (red) separate from control populations (blue) based on their methylation profiles. **(C)** MVP scores for DBA5 cases (blue) and control samples (gray), with other DBAS cohorts (DBA1, DBA4, DBA7, DBA6, and DBA10) included as testing sets (gray circles). The abbreviations for the disorders indicated on X-axis are listed in Supplemental Table 5.

**(D)** MVP score distribution for DBA5 cases, revealing relatively high MVP scores in some DBA5 samples.


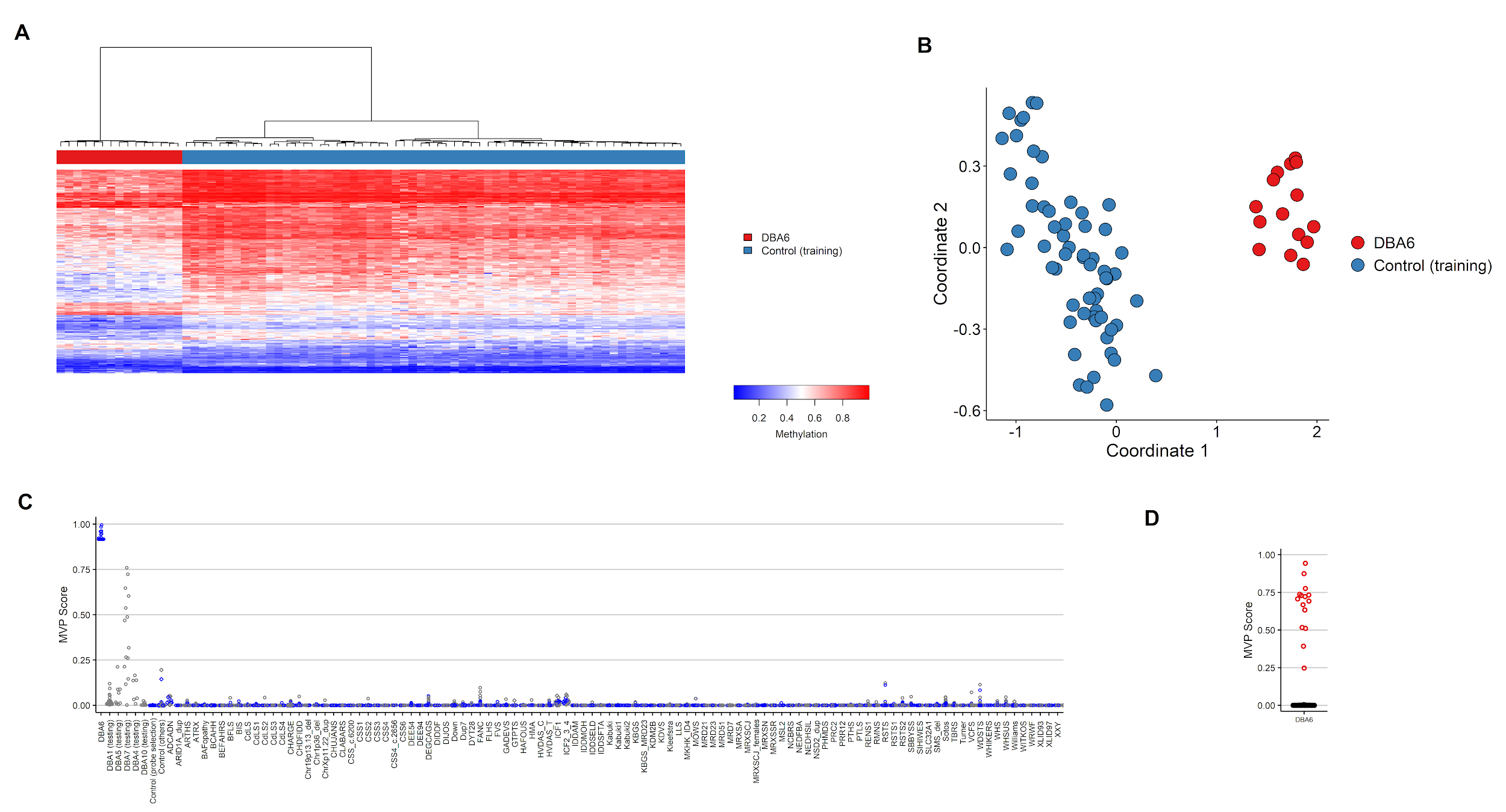


**Figure S6.** **Identification of an episignature in the DBA6 cohort. (A)** Heatmap revealing the distinct separation of methylation profiles between DBA6 cases (red, n=15) and control samples (blue). **(B)** MDS plot showing the clustering of DBA6 cases (red) and control populations (blue) based on their methylation profiles. **(C)** MVP scores for DBA6 cases (blue) and control samples (gray), with other DBAS cohorts (DBA1, DBA4, DBA5, DBA7, and DBA10) included as testing sets (gray circles). The abbreviations for the disorders indicated on X-axis are listed in Supplemental Table 5. **(D)** MVP score distribution for DBA6 cases, indicating high MVP scores in most DBA6 sa mples.

**
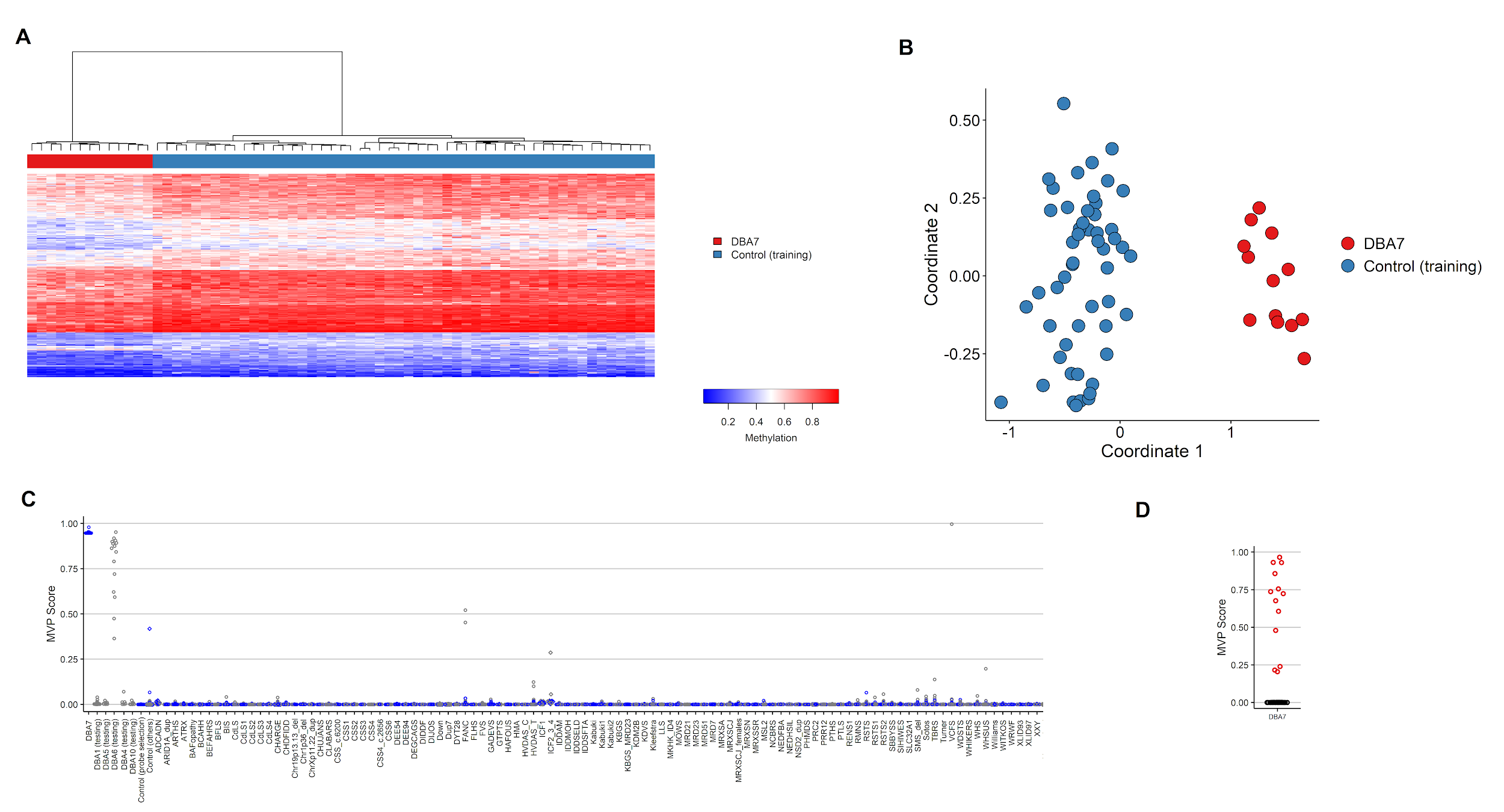
**

**Figure S7. Identification of an episignature in the DBA7 cohort. (A)** Heatmap depicting the distinct methylation patterns between DBA7 cases (red, n=13) and control samples (blue). **(B)** MDS plot showing the clear separation of DBA7 cases (red) from control populations (blue) based on their methylation profiles. **(C)** MVP scores for DBA7 cases (blue) and control samples (gray), with other DBAS cohorts (DBA1, DBA5, DBA6, DBA4, and DBA10) used as testing sets (gray circles). The abbreviations for the disorders indicated on X-axis are listed in Supplemental Table 5. **(D)** MVP score distribution for DBA7 cases, indicating high MVP scores in most DBA7 samples.

**
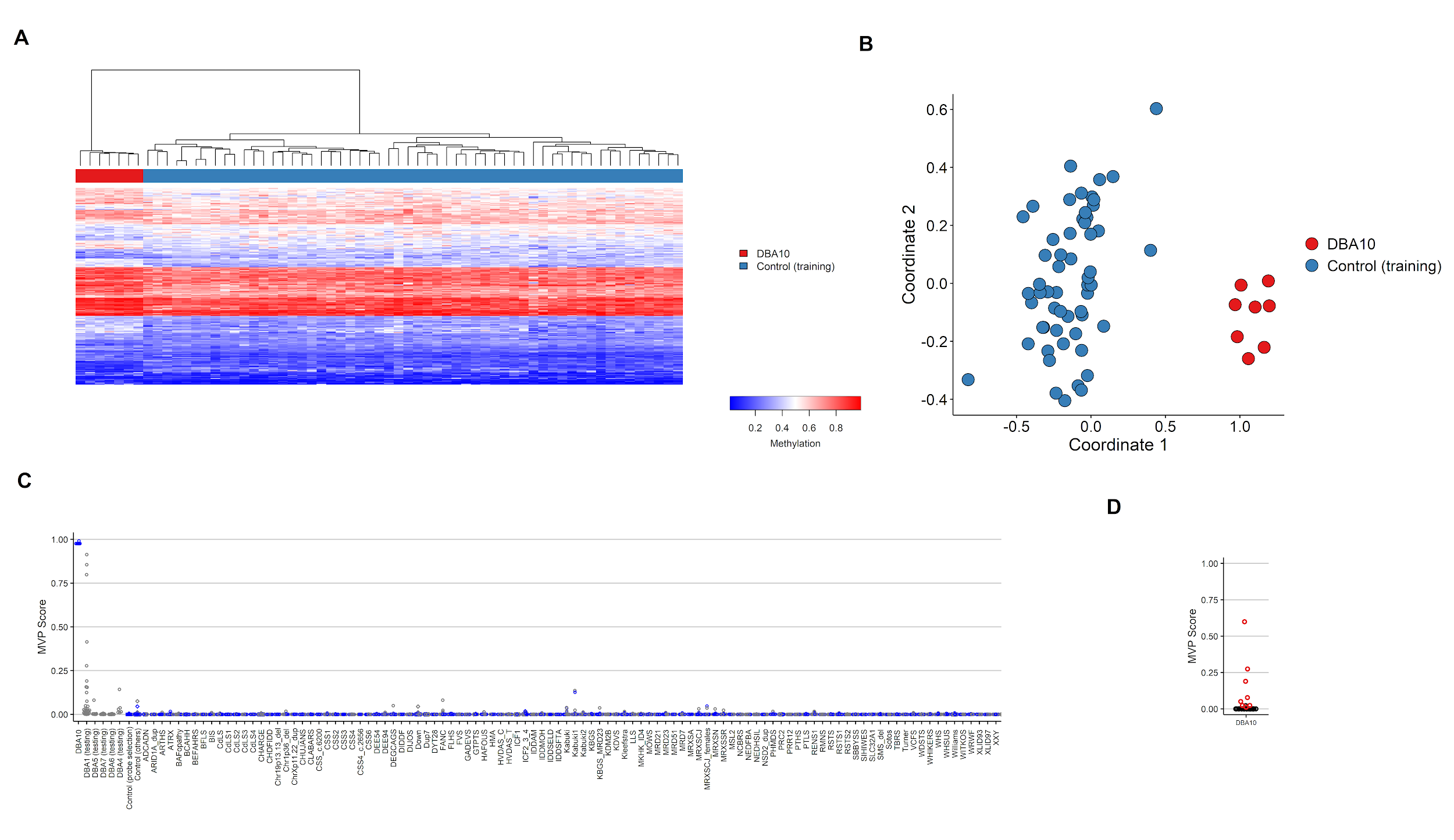
**

**Figure S8.** **Detection of an episignature in the DBA10 cohort**. **(A)** Heatmap highlighting the distinct methylation profiles of DBA10 cases (red, n=8) compared to control samples (blue). **(B)** MDS plot showing the clear separation between DBA10 cases (red) and control samples (blue) based on their methylation profiles. **(C)** MVP scores for DBA10 cases (blue) and control samples (gray), with other DBAS cohorts (DBA1, DBA5, DBA7, DBA6, and DBA4) included as testing sets (gray circles). The abbreviations for the disorders indicated on X-axis are listed in Supplemental Table 5. **(D)** MVP score distribution for DBA10 cases, with most DBA10 samples showing low MVP scores.

**
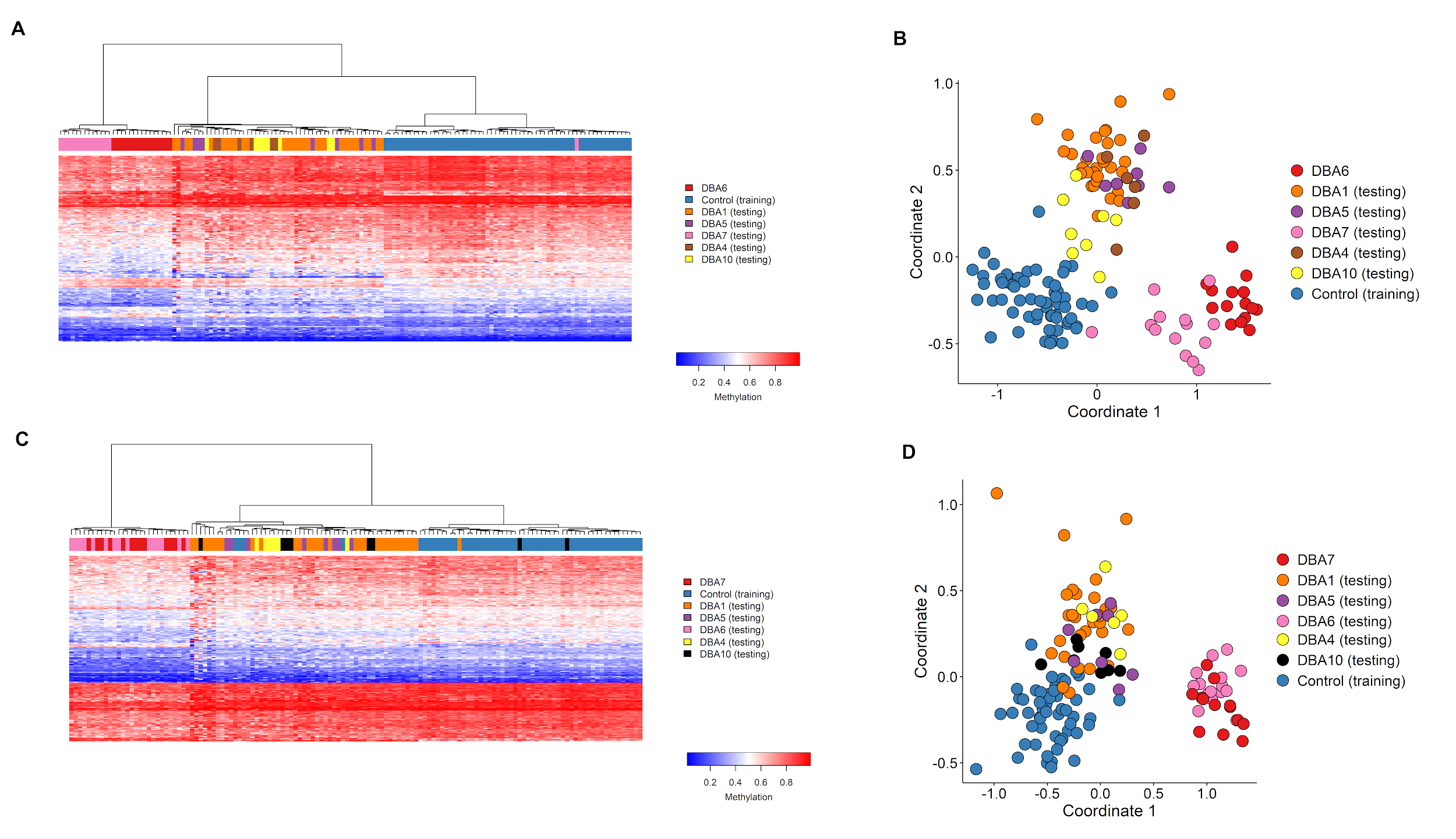
**

**Figure S9. Evaluation of the episignature across multiple DBAS cohorts. (A)** Heatmap showing the separation of methylation profiles between DBA6 cases (red), control samples (blue), and samples from different DBAS cohorts (DBA1, DBA5, DBA7, DBA4, DBA10) used for testing (colored boxes). **(B)** MDS plot illustrating the clustering of DBA6 cases (red), control samples (blue), and DBAS testing cohorts (DBA1 - orange, DBA5 - purple, DBA7 - pink, DBA4 - brown, DBA10 - yelow), based on their methylation profiles. **(C)** Heatmap showing the separation of methylation profiles between DBA7 cases (red), control samples (blue), and samples from other DBAS cohorts (DBA1, DBA5, DBA6, DBA4, DBA10) used for testing (colored boxes). **(D)** MDS plot showing the clustering of DBA7 cases (red), control samples (blue), and DBAS testing cohorts (DBA1 - orange, DBA5 - purple, DBA6 - pink, DBA4 - yellow, DBA10 - black), based on their methylation profiles.


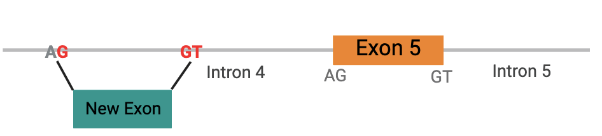

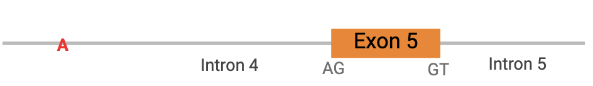

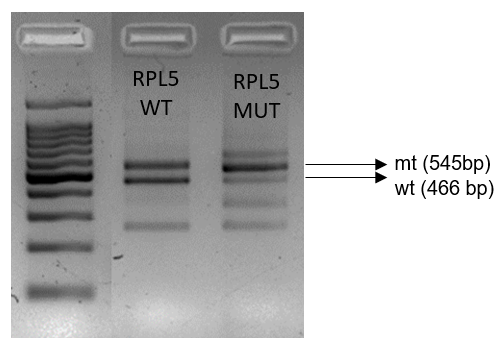

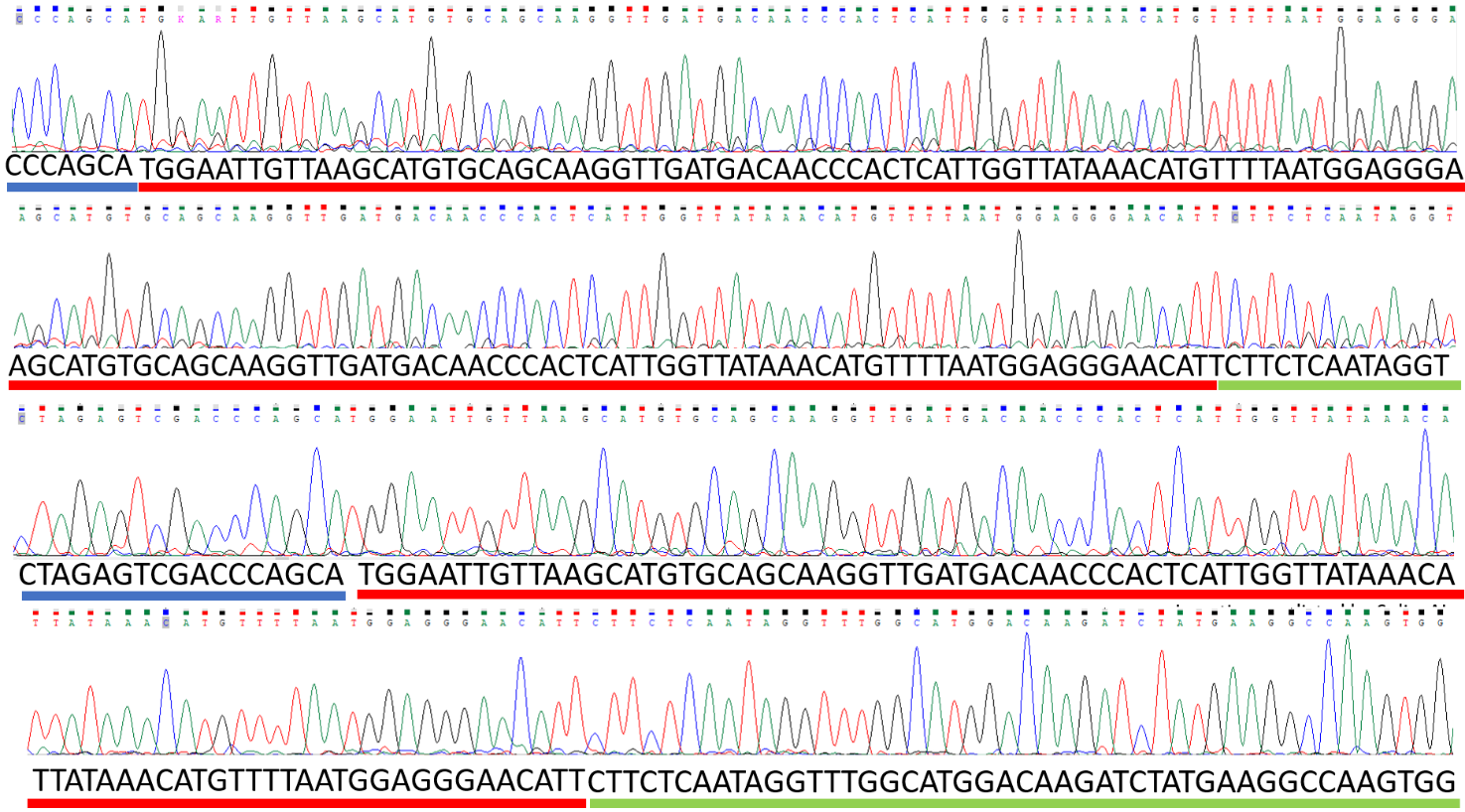


**A**

**B**

**C**

**D**

**D**


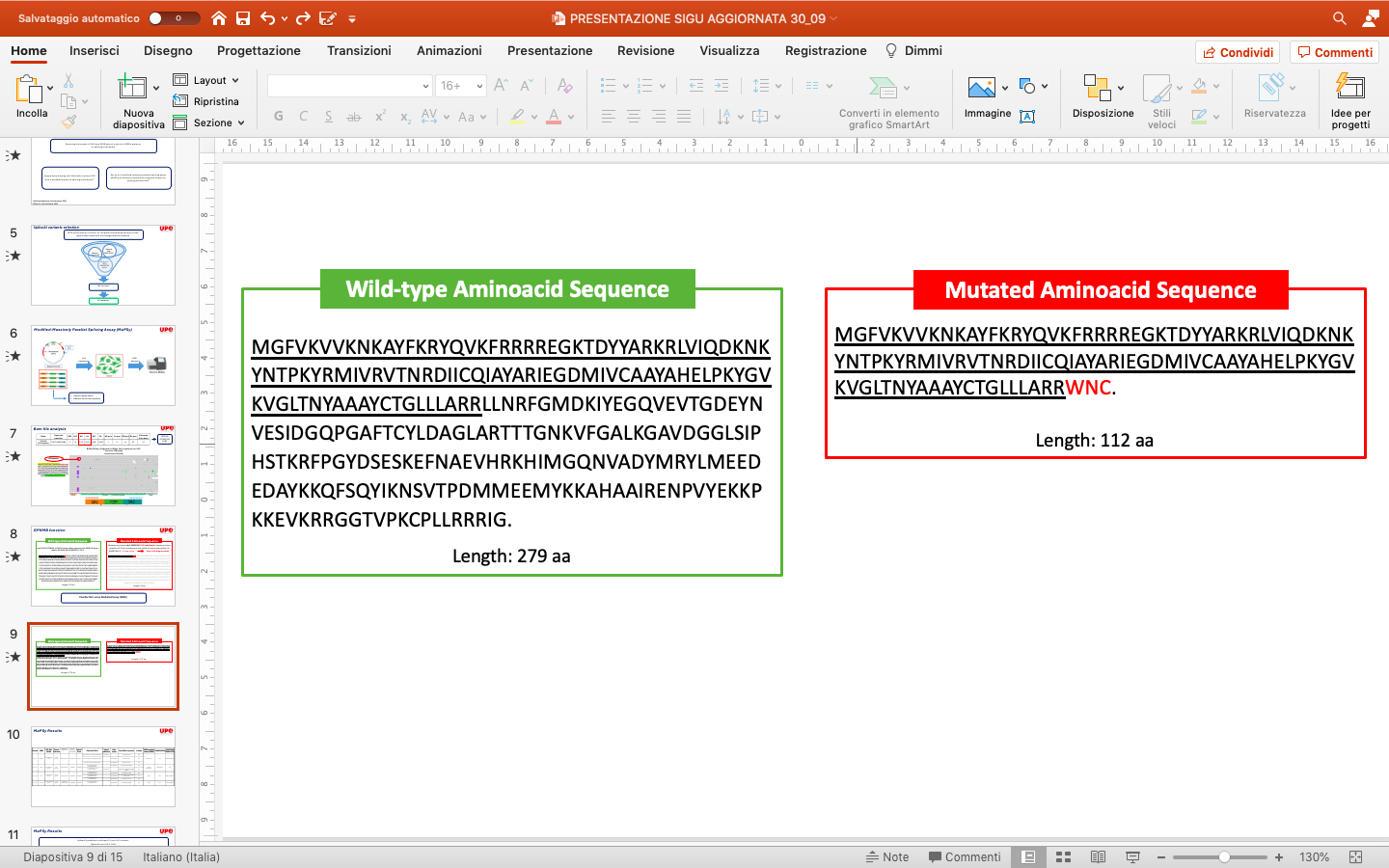


**Figure S10. Functional characterization of the *RPL5*: c.325-380A>G: p.?**

**(A)** left panel: representation of wildtype sequence of *RPL5* c.325-380A; right panel: representation of mutated sequence of *RPL5* c.325-380G. **(B)** PCR performed on cDNA using SD6/SA2. **(C)** Representation of the 79 bp insertion caused by mutant c.325-380G. Colour legend: blue, exon SD6; red, insertion predicted by SpliceAI; green, exon 5 *RPL5*. **(D)** Depicts the wildtype amino acid sequence and the mutated amino acid sequence. The wildtype sequence consists of 279 residues. The insertion of 79 bp results in a frameshift mutation altering three amino acids (LLN to WNC) and introducing a premature stop codon at position 112. Consequently, this leads to the production of a truncated protein, likely targeted for nonsense-mediated decay. Legend: LLN = Leucine – Leucine – Asparagine, WNC = Tryptophan – Asparagine – Cysteine.


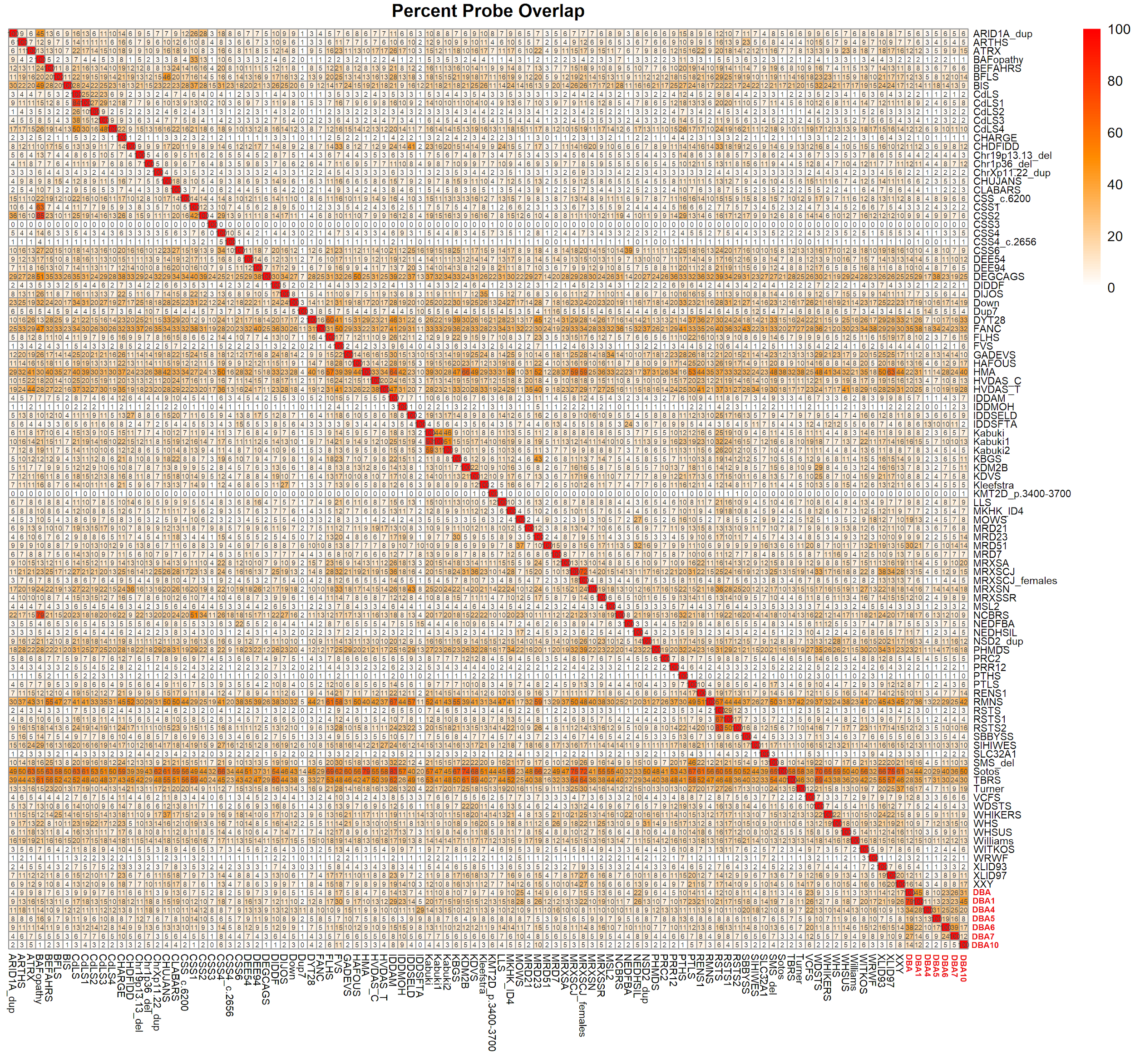


**Figure S11. Percent Probe Overlap Across DBAS and Control Cohorts**
Heatmap showing the percentage of probe overlap between different DBAS cohorts (DBA1, DBA4, DBA5, DBA6, DBA7, DBA10) and control samples. The intensity of the colour represents the degree of overlap, with darker red indicating higher overlap. Samples from DBAS cohorts and control groups are displayed along both axes, with DBAS cohorts highlighted in red for easy identification.

**
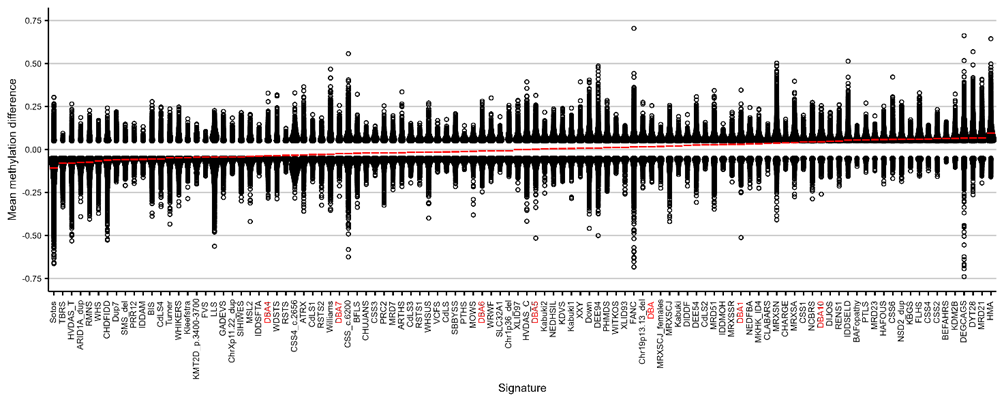
**

**Figure S12. Methylation differences across DBAS cohorts.** Mean methylation levels (red lines) and individual differentially methylated probes (circles) are shown for each cohort. An overall hypermethylation trend was observed in the entire DBAS cohort, DBA1, and DBA10, while DBA4, DBA7, DBA6, and DBA5 exhibited a hypomethylation trend.

**
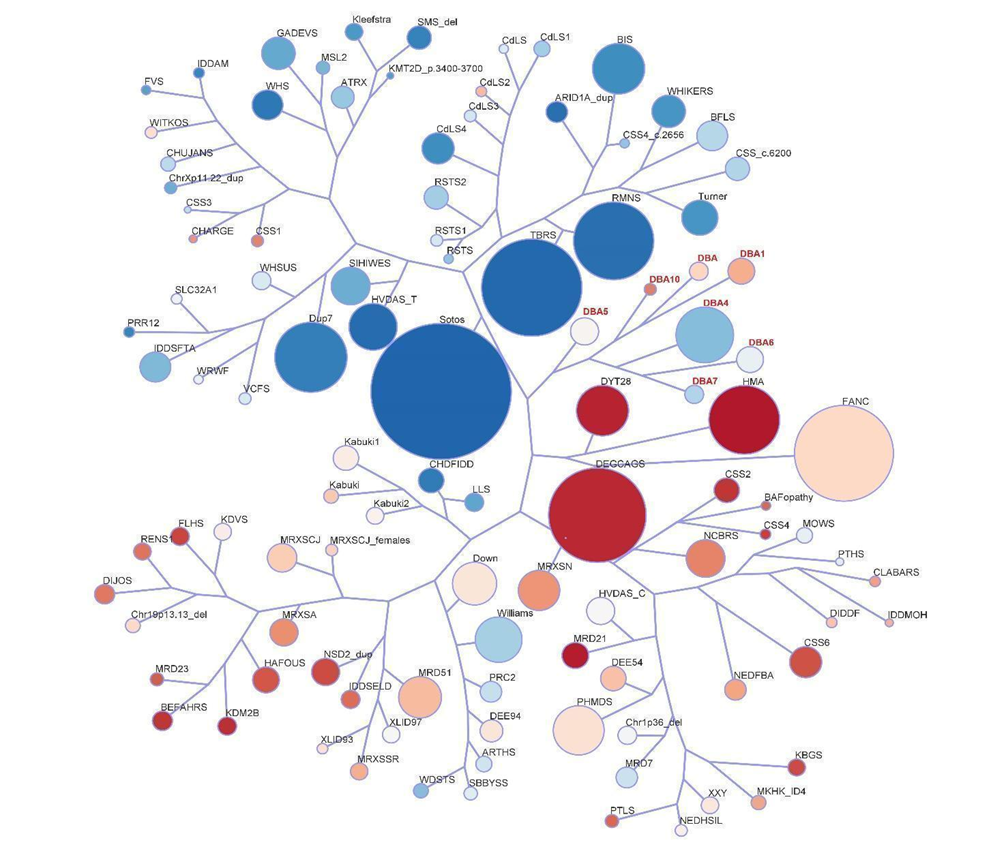
**

**Figure S13. Functional correlation (Tree and Leaf diagram) of DBAS subtypes relative to 99 other cohorts from the EKD.** The visualization was generated using Euclidean clustering based on the top 500 DMPs for each cohort. Cohort samples were aggregated by the median methylation values of each DMP within the group. In the diagram, each leaf node represents a specific cohort, with node sizes reflecting the total number of DMPs, and the node colors indicate the mean methylation difference. All DBAS subtypes clustered closely together, while separate branches were observed for subtypes exhibiting hypomethylated or hypermethylated trends. Abbreviations for the listed epigenetic profiles are listed in table S5.

**
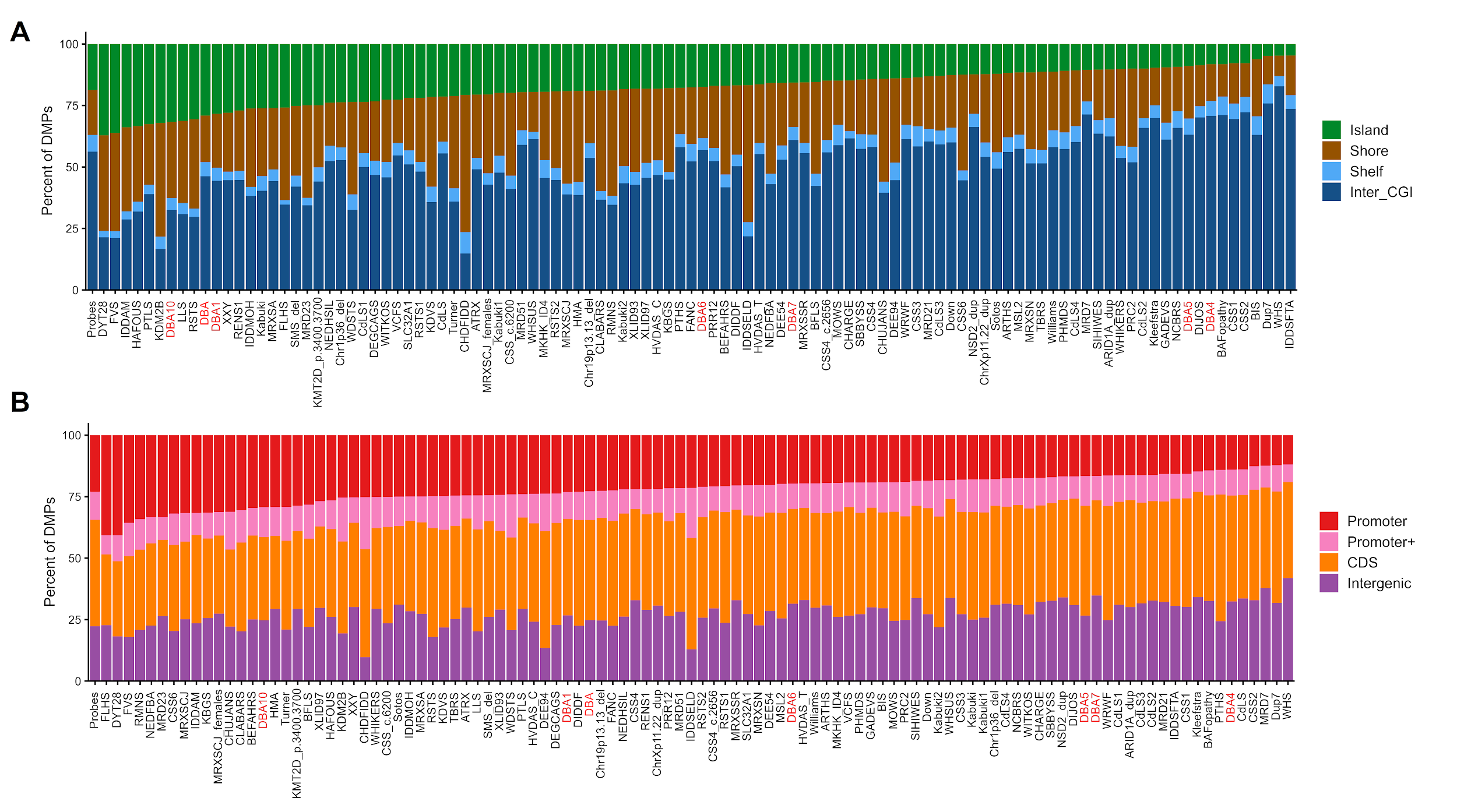
**

**Figure S14. Distribution of Differentially Methylated probes by Genomic Feature.** **(A)** Bar plot showing the distribution of DMPs across different genomic regions: Island (green), Shore (brown), Shelf (light blue), and Intergenic CGI (blue). DMPs from DBAS cohorts (DBA10, DBA, DBA1, DBA6, DBA7, DBA5, and DBA4) are highlighted in red. **(B)** Bar plot displaying the distribution of DMPs across different genomic features: Promoter (red), Promoter+ (pink), CDS (orange), and Intergenic (purple). DBAS cohorts are marked in red for easy identification.
